# The Role of Mammalian Target of Rapamycin (mTOR) and Adenosine Monophosphate-Activated Protein Kinase (AMPK) Signaling in Skeletal Muscle Hypertrophy: A Literature Review With Implications for Health and Disease

**DOI:** 10.7759/cureus.96018

**Published:** 2025-11-03

**Authors:** Seon Yeop Jeong

**Affiliations:** 1 Primary Care, MaxHealth, Bradenton, USA

**Keywords:** amino acids, ampk, autophagy, fasting, mitochondrial biogenesis, mtor, muscle hypertrophy, protein synthesis, resistance exercise

## Abstract

Maintaining skeletal muscle mass is fundamental not only for strength and mobility but also for metabolic health, disease prevention, and healthy aging. Skeletal muscle functions as a dynamic metabolic organ, and its maintenance depends on the delicate equilibrium between mammalian target of rapamycin (mTOR), which drives anabolic processes, such as protein synthesis and hypertrophy, and adenosine monophosphate-activated protein kinase (AMPK), which promotes catabolic renewal through mitochondrial biogenesis, autophagy, and energy conservation. Sustained mTOR hyperactivation contributes to obesity, insulin resistance, neurodegeneration, and cancer, while AMPK activation counterbalances these effects by restoring cellular energy balance and enhancing metabolic resilience. Thus, optimal muscle and metabolic health depend not on dominance of one pathway, but on the strategic balance between mTOR and AMPK activity.

Human trials and meta-analyses show that for individuals engaged in resistance training, a total protein intake of approximately 1.6 g/kg/day effectively maximizes muscle protein synthesis, with diminishing returns beyond that level. Distributing protein evenly across meals enhances anabolic efficiency, about 0.25 g/kg per meal in younger adults and 0.40 g/kg per meal in older adults, which helps overcome age-related anabolic resistance. Protein quality is equally critical: leucine-rich, rapidly absorbed sources such as whey elicit the most robust mTOR activation and muscle-building response.

Exercise mode and energy timing further shape this anabolic-catabolic balance. Concurrent endurance and resistance training may attenuate hypertrophy depending on session order, intensity, and training status, whereas intermittent fasting and time-restricted feeding improve body composition and cardiometabolic health when total energy and protein needs are still met. Together, these findings suggest that integrating structured resistance exercise, well-distributed, high-quality protein intake, and periodic AMPK activation through fasting or aerobic training provides a practical framework for sustaining muscle mass, optimizing metabolic function, and promoting longevity.

## Introduction and background

Skeletal muscle metabolism is intricately regulated by the interplay between two pivotal cellular pathways: the mammalian target of rapamycin (mTOR) and adenosine monophosphate-activated protein kinase (AMPK). mTOR serves as a central modulator of protein synthesis and muscle growth, responding to stimuli such as amino acids, growth factors, and resistance exercise [1]. Conversely, AMPK acts as an energy sensor, activated during states of energy deficit, such as fasting, nutrient restriction, or prolonged/intense exercise, to promote catabolic processes like autophagy and mitochondrial biogenesis, thereby conserving energy and maintaining cellular homeostasis [2].

These pathways exhibit antagonistic effects. While mTOR is crucial for muscle hypertrophy, its chronic hyperactivation has been implicated in diseases such as type 2 diabetes, cancer, and neurodegenerative conditions [1]. AMPK inhibits mTOR, and its activation may help mitigate these pathologies [3]. However, given mTOR’s essential role in cell proliferation and growth, a balanced regulation of mTOR and AMPK is likely optimal for overall health. Accordingly, this narrative review aims to examine strategies for developing and maintaining skeletal muscle mass and promoting longevity through the modulation of mTOR and AMPK, while also highlighting diseases associated with dysregulation of these pathways.

Methodological note

This work is a narrative review based on a literature search conducted through PubMed, Scopus, and Google Scholar, encompassing articles published up to April 2025. Keywords included mTOR, AMPK, protein intake, resistance training, intermittent fasting (IF), and longevity. Both human and relevant mechanistic studies were considered to provide a balanced synthesis of current evidence. Formal inclusion/exclusion criteria and quantitative risk-of-bias assessments were not applied, consistent with the narrative design.

## Review

Function of mTOR signaling pathway

The mTOR signaling pathway is a central regulator of cell growth and proliferation. Its activation triggers cascades that lead to both hyperplasia (increase in cell number) and hypertrophy (increase in cell size) [1]. The key components initiating these cellular changes are mTORC1 and mTORC2, two distinct protein complexes within the mTOR pathway [1].

Role of mTORC1

**Figure 1:**
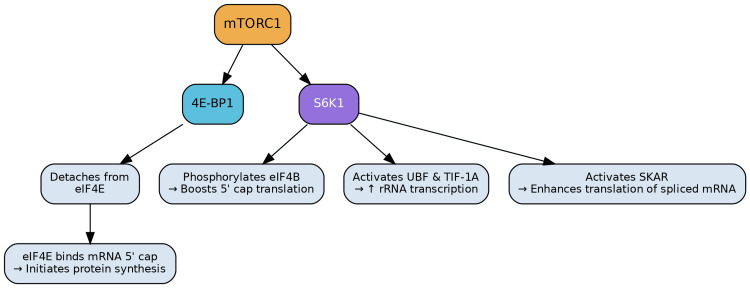
Role of mTORC1 in Protein Metabolism mTORC1 promotes anabolic processes by phosphorylating its downstream effectors, including 4E-BP1 and S6K1, to enhance protein synthesis and ribosomal biogenesis. Phosphorylation of 4E-BP1 releases eIF4E to initiate cap-dependent translation, while activated S6K1 stimulates eIF4B, UBF, TIF-1A, and SKAR, thereby augmenting mRNA translation, rRNA transcription, and spliced transcript translation. Image credits: Figure created by the author using information from [1,4,5].

The mammalian target of rapamycin complex 1 (mTORC1) serves as a central orchestrator of anabolic processes within the cell, primarily by promoting protein synthesis. This is largely achieved through the phosphorylation of two key downstream effectors: eukaryotic initiation factor 4E-binding proteins (4E-BPs) and p70 S6 Kinase 1 (S6K1). Unphosphorylated 4E-BP1 sequesters eukaryotic translation initiation factor 4E (eIF4E), thereby inhibiting cap-dependent mRNA translation. Upon phosphorylation by mTORC1, 4E-BP1 releases eIF4E, enabling it to bind to the mRNA 5' cap and initiate protein synthesis [4,5]. Concurrently, mTORC1-mediated phosphorylation of S6K1 further augments protein synthesis by activating eIF4B, which also promotes 5' cap-dependent translation. S6K1 additionally upregulates upstream binding factor (UBF) and transcription initiation factor 1A (TIF-1A), consequently boosting RNA polymerase I activity and ribosomal RNA (rRNA) transcription. Furthermore, S6K1 activates the specific target of S6 kinase 1 (SKAR), enhancing the translation of spliced transcripts (Figure 1) [1].

**Figure 2:**
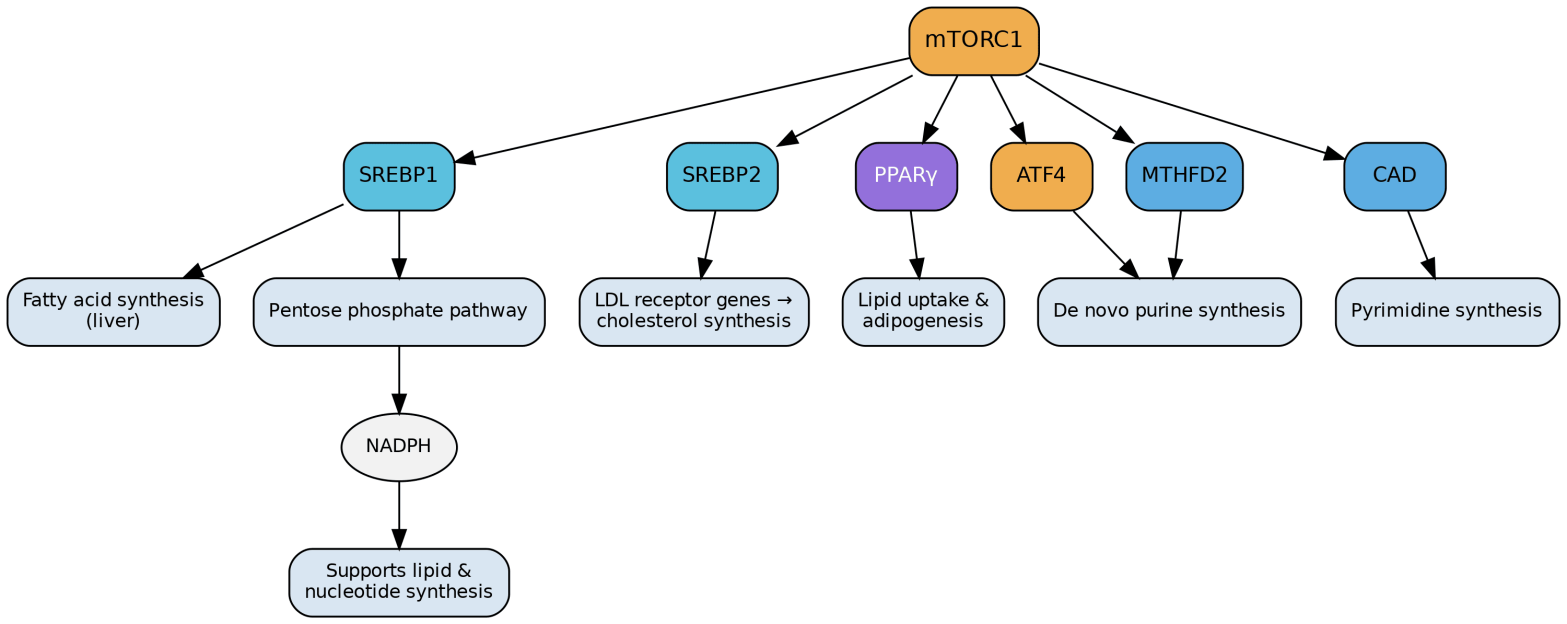
Role of mTORC1 in Lipid and Nucleotide Metabolism Beyond protein synthesis, mTORC1 regulates lipid and nucleotide biosynthesis to support cell growth and proliferation. By activating SREBP1 and SREBP2, mTORC1 promotes fatty acid synthesis in the liver and cholesterol synthesis through LDL receptor upregulation. PPARγ further enhances lipid uptake and adipogenesis. In parallel, SREBP activation stimulates the pentose phosphate pathway, generating NADPH for lipid and nucleotide production. mTORC1 also facilitates nucleotide synthesis by activating ATF4 and MTHFD2 for purine biosynthesis and by upregulating CAD, a key enzyme complex for pyrimidine biosynthesis. Image credits: Figure created by the author using information from [1,6-8].

Beyond protein synthesis, mTORC1 significantly influences lipid synthesis. It achieves this by increasing the activity of sterol regulatory element-binding protein 1/2 (SREBP1/2) and peroxisome proliferator-activated receptor-gamma (PPARγ). Specifically, SREBP1 activates genes responsible for fatty acid synthesis in the liver, while SREBP2 upregulates LDL receptor genes to promote cholesterol synthesis [6]. PPARγ, in turn, enhances gene expression linked to increased lipid uptake and adipogenesis. Moreover, SREBP activation stimulates the pentose phosphate pathway, providing crucial NADPH for both lipid and nucleotide biosynthesis (Figure 2) [1].

mTORC1 also plays a vital role in facilitating nucleotide synthesis, particularly in proliferating cells that require abundant building blocks for DNA replication and rRNA production. mTORC1 achieves this by activating the transcription factor ATF4 and the mitochondrial tetrahydrofolate cycle enzyme methylenetetrahydrofolate dehydrogenase 2 (MTHFD2), both of which are critical for de novo purine synthesis [7]. Additionally, mTORC1 activates carbamoyl-phosphate synthetase 2, aspartate transcarbamoylase, dihydroorotase (CAD), a multi-enzyme complex essential for pyrimidine synthesis (Figure 2) [8].

**Figure 3:**
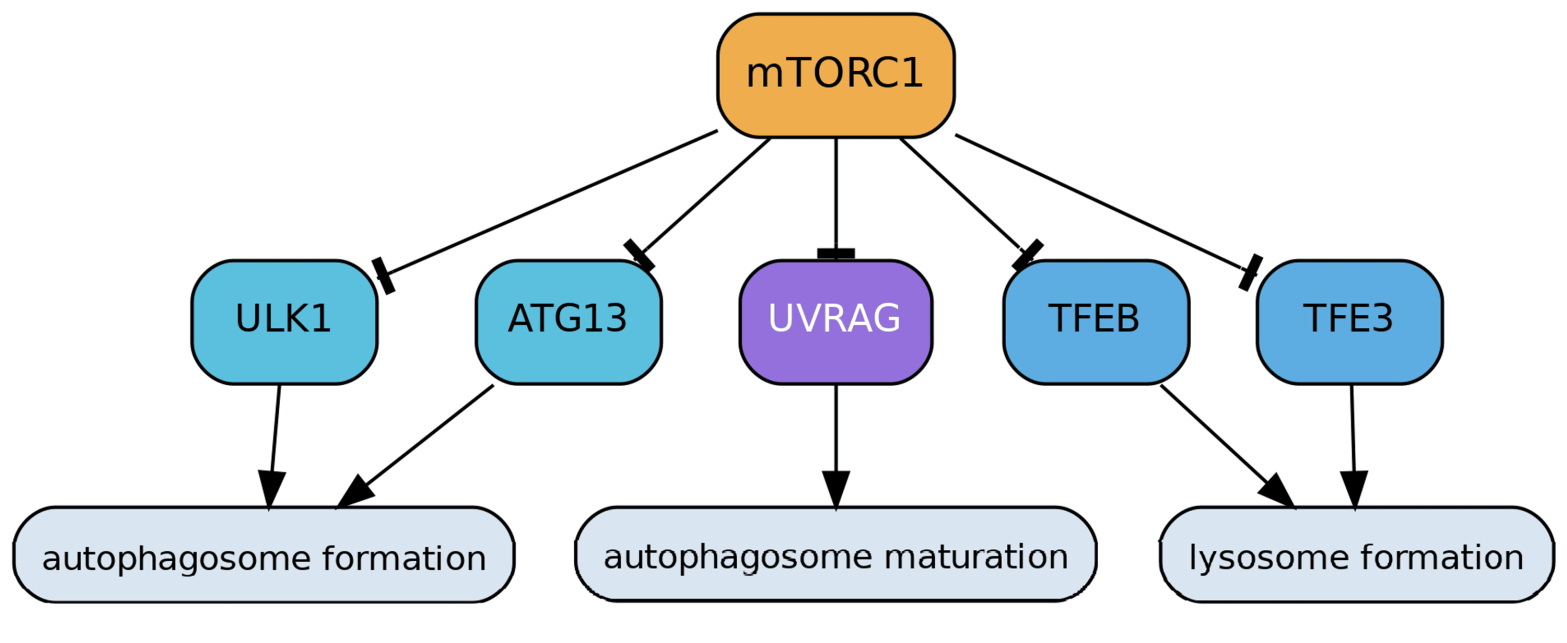
mTORC1 Regulation of Autophagy mTORC1 acts as a central negative regulator of autophagy, suppressing lysosomal-dependent degradation and recycling pathways. It directly phosphorylates ULK1 and ATG13, preventing autophagosome initiation. mTORC1 also phosphorylates UVRAG, thereby impairing autophagosome maturation. In addition, by inhibiting the nuclear translocation of transcription factors TFEB and TFE3, mTORC1 blocks the expression of lysosomal biogenesis genes, ultimately disrupting lysosome formation and reducing the cell’s degradative capacity. Image credits: Figure created by the author using information from [1,3,9,10].

Conversely, mTORC1 acts as a potent inhibitor of autophagy, a crucial lysosomal-dependent catabolic process responsible for degrading and recycling damaged or unnecessary cellular components. mTORC1 directly downregulates autophagy by applying inhibitory phosphorylation to key initiating regulators, Unc-51-like autophagy-activating kinase 1 (ULK1) and autophagy-related protein 13 (ATG13) [3]. This phosphorylation prevents their collaborative function in forming the autophagosome, leading to the potential accumulation of cellular debris. Furthermore, mTORC1 interferes with autophagosome maturation by phosphorylating UV radiation resistance-associated gene protein (UVRAG), thereby further suppressing effective autophagy [9]. Lastly, mTORC1 inhibits the nuclear translocation and activity of transcription factors EB (TFEB) and E3 (TFE3), which are vital for enhancing the expression of genes involved in lysosomal biogenesis and autophagy. This inhibition disrupts lysosome formation, impairing the cell's capacity to degrade redundant components and contributing to cellular dysfunction (Figure 3) [10].

Role of mTORC2

**Figure 4:**
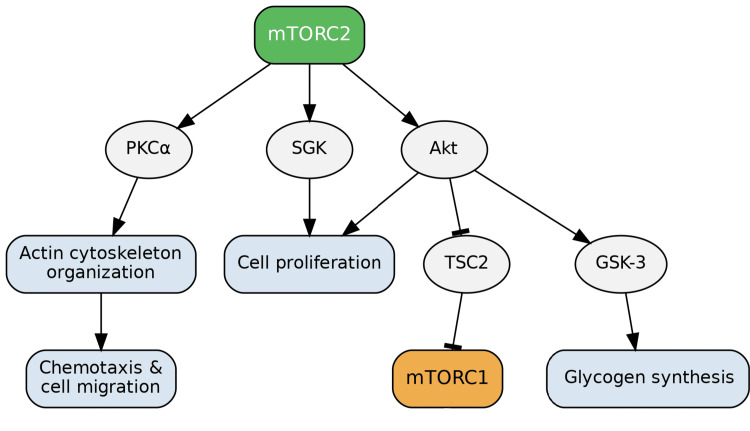
Targets of mTORC2 Signaling mTORC2 regulates multiple cellular processes by activating downstream effectors, including Akt, PKC, and SGK. Akt promotes cell survival by phosphorylating BAD, preventing apoptosis and driving cell cycle progression. It also enhances glucose metabolism through phosphorylation of GSK-3, leading to glycogen synthesis, and indirectly stimulates mTORC1 activity by inhibiting TSC2, thereby contributing to autophagy suppression. PKCα, another mTORC2 target, influences cytoskeletal organization and actin filament assembly, which are essential for chemotaxis and cell migration. Inhibition of mTORC2 disrupts these cytoskeletal processes, reducing cell motility. Image credits: Figure created by the author using information from [1,11-15].

While less comprehensively understood than mTORC1, mTORC2 plays critical roles in regulating the cytoskeleton, cellular metabolism, and cell survival. mTORC2 exerts its effects primarily by activating Akt (also known as protein kinase B), protein kinase C (PKC), and serum- and glucocorticoid-induced protein kinase (SGK) [1]. Akt is a central effector involved in cell survival, cell cycle progression, glucose metabolism, and autophagy. Through its pro-survival functions, Akt phosphorylates BAD, causing it to dissociate from the Bcl2/Bcl-X complex and thus preventing apoptosis [11]. Akt also promotes cell proliferation by inducing an exit from the G1-S phase cell cycle arrest. In terms of metabolism, Akt phosphorylates glycogen synthase kinase 3 (GSK-3), thereby promoting glycogen synthesis. Furthermore, Akt indirectly enhances mTORC1 activity by inhibiting tuberous sclerosis complex (TSC2) 2, a major negative regulator of mTORC1. This Akt-mediated upregulation of mTORC1, in turn, contributes to the downregulation of autophagy. Consequently, the activation of Akt by mTORC2 significantly promotes cell survival and proliferation [12]. Regarding the cytoskeleton, Protein kinase Cα (PKCα), a known target of mTORC2, is thought to be involved in its regulation, though the comprehensive mechanism remains under investigation [13]. Stimulation of PKCα has been observed to induce the organization of the actin cytoskeleton. Consistent with this role, inhibition of mTORC2 diminishes processes like chemotaxis and cell migration, which results from impaired assembly of the actin cytoskeleton network (Figure 4) [14,15].

Function of AMPK signaling pathway

**Figure 5:**
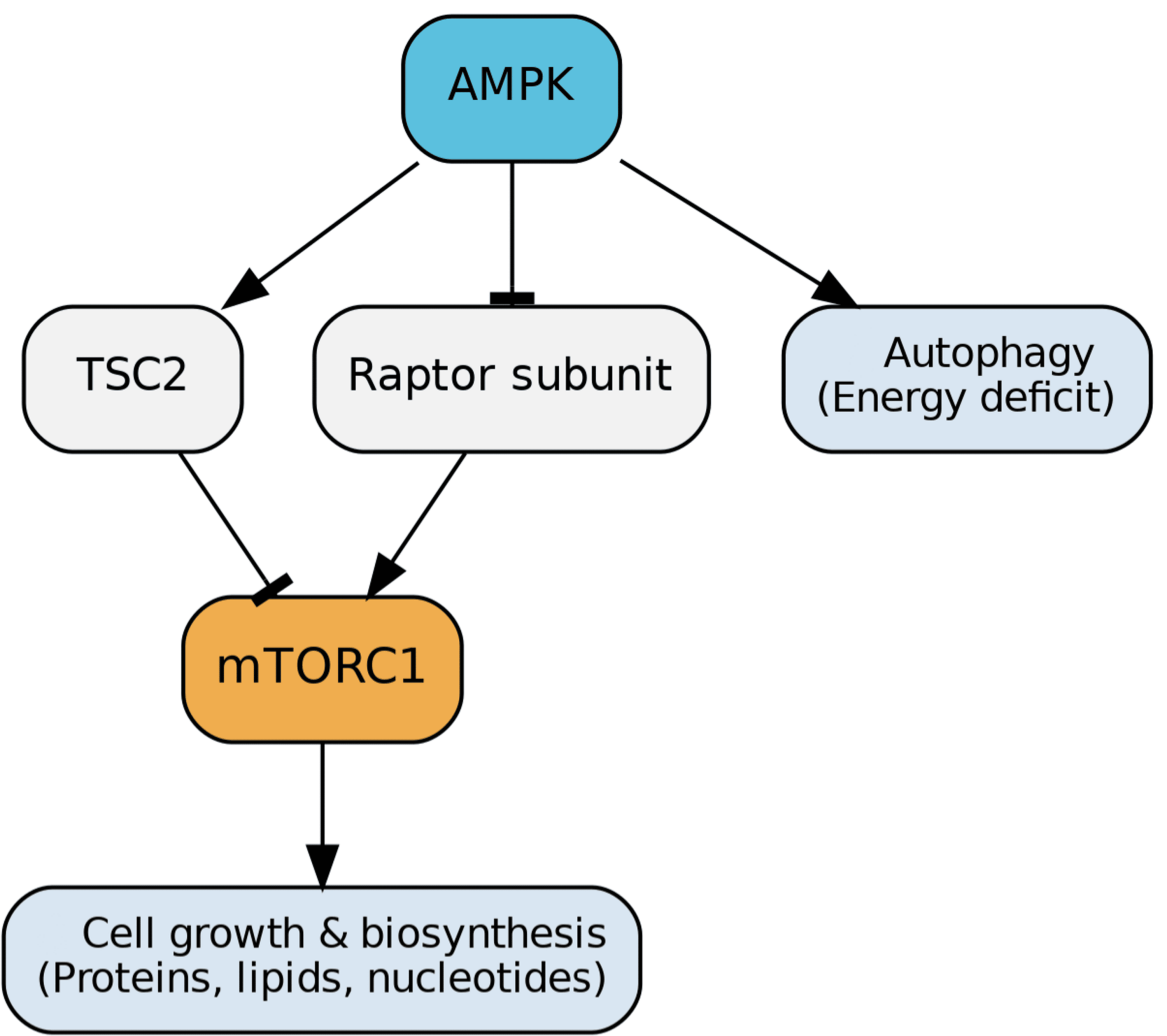
AMPK Regulation of mTORC1 and Autophagy AMPK serves as a master regulator of cellular energy balance by sensing energy stress and activating catabolic pathways. Under conditions of low energy availability, AMPK promotes autophagy by upregulating TSC2, an upstream inhibitor of mTORC1, and by directly phosphorylating Raptor, a key mTORC1 subunit. Through these mechanisms, AMPK suppresses mTORC1 activity, thereby limiting cell growth and reducing the biosynthesis of proteins, lipids, and nucleotides. This shift conserves energy while maintaining cellular homeostasis. Image credits: Figure created by the author using information from [1,2].

AMPK functions as a primary cellular energy sensor and a critical regulator of cell growth and autophagy. It plays crucial roles in modulating glucose and lipid metabolism, as well as maintaining mitochondrial homeostasis [1]. During states of energy deficit, AMPK regulates cell growth and proliferation by promoting autophagy, a process that involves the engulfment and breakdown of cellular components. AMPK facilitates this by indirectly suppressing the mTORC1 pathway through the upregulation of TSC2 activity, a major inhibitor of mTORC1. Additionally, AMPK directly inhibits mTORC1 by phosphorylating its subunit Raptor. This collective action limits cell growth and reduces the biosynthesis of proteins, lipids, and nucleotides (Figure 5) [2].

AMPK is a pivotal activator of autophagy. It directly upregulates this process by phosphorylating multiple sites on ULK1. While ULK1 is known to be inhibited by mTORC1, AMPK's suppressive effect on mTORC1 indirectly disinhibits ULK1, further promoting autophagosome formation [16,17].

**Figure 6:**
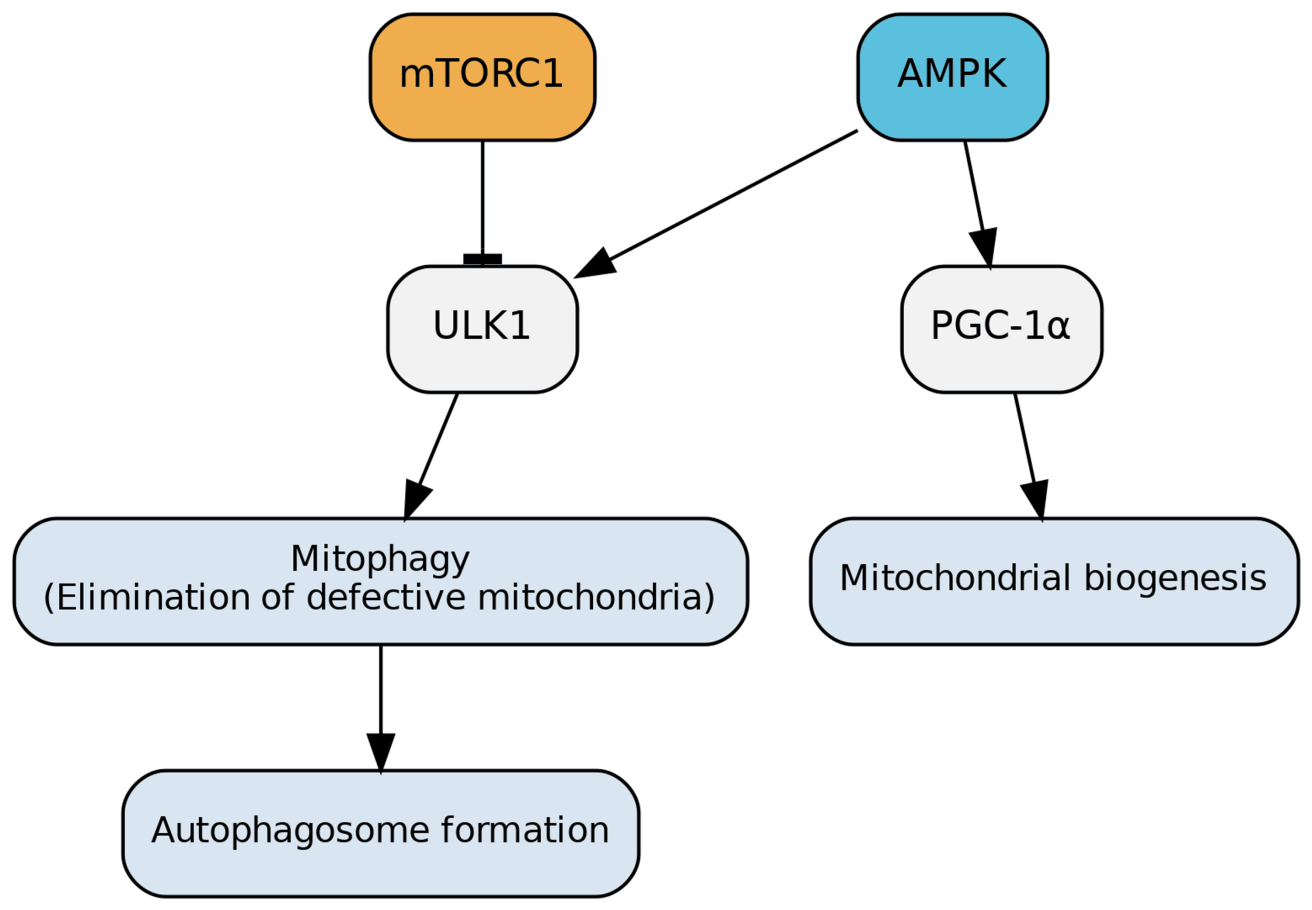
Role of AMPK in Autophagy and Mitochondrial Biogenesis AMPK directly promotes autophagy by phosphorylating multiple sites on ULK1. By suppressing mTORC1, AMPK also indirectly relieves ULK1 inhibition, further enhancing autophagosome formation. In addition to autophagy, AMPK plays a critical role in mitochondrial quality control. It induces mitophagy through ULK1 activation to eliminate defective mitochondria, while simultaneously stimulating mitochondrial biogenesis via PGC-1α-dependent transcription. Genetic defects in AMPK result in impaired mitochondrial turnover and accumulation of dysfunctional mitochondria, underscoring its role in maintaining cellular health. Image credits: Figure created by the author using information from [2,16,17].

Furthermore, AMPK is central to maintaining mitochondrial homeostasis. Its stimulation of ULK1 triggers the selective elimination of defective mitochondria (mitophagy), while simultaneously inducing new mitochondrial biogenesis through the activation of peroxisome proliferator-activated receptor gamma coactivator 1-alpha (PGC-1α)-dependent transcription. The importance of AMPK in mitochondrial control is underscored by findings that genetic defects in AMPK genes lead to a notable accumulation of defective mitochondria, demonstrating a substantial effect on cell populations, such as hematopoietic stem cells (Figure 6) [2].

**Figure 7:**
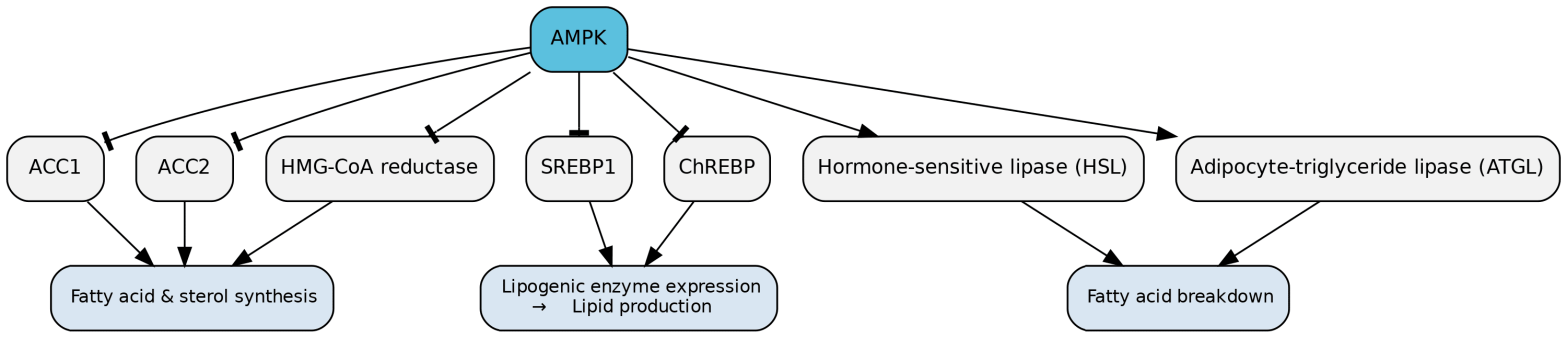
Role of AMPK in Lipid Metabolism AMPK functions as a key regulator of lipid homeostasis by suppressing lipogenesis and promoting lipid catabolism. It acutely inhibits fatty-acid and sterol synthesis through phosphorylation of the rate-limiting enzymes ACC1, ACC2, and HMG-CoA reductase. Over the long term, AMPK downregulates lipogenic gene expression by phosphorylating and inhibiting SREBP1 and ChREBP, two transcription factors that enhance lipogenesis. In adipose tissue, AMPK further promotes fatty acid breakdown by directly activating hormone-sensitive lipase (HSL) and adipocyte-triglyceride lipase (ATGL), thereby facilitating lipid catabolism. Image credits: Figure created by the author using information from [2,18-21].

AMPK also plays a critical role in regulating lipid synthesis. It acutely suppresses fatty-acid and sterol synthesis by phosphorylating rate-limiting enzymes, specifically ascetyl-CoA carboxylase 1 (ACC1) and ACC2, and 3-hydroxy-3-methylglutaryl-CoA reductase (HMG-CoA reductase), respectively [18]. In addition to these acute effects, AMPK exerts a long-term inhibitory effect on lipogenesis by phosphorylating SREBP1, which activates genes required for lipogenesis; this inhibitory phosphorylation leads to a decrease in lipogenic enzyme expression. AMPK also phosphorylates the glucose-sensitive transcription factor ChREBP (carbohydrate response-element-binding protein), another enhancer of lipogenic enzyme expression, thereby dampening lipid production [19]. Moreover, in adipose tissue, AMPK promotes fatty acid catabolism by directly phosphorylating and activating hormone-sensitive lipase (HSL) and adipocyte-triglyceride lipase (ATGL) (Figure 7) [20,21].

**Figure 8:**
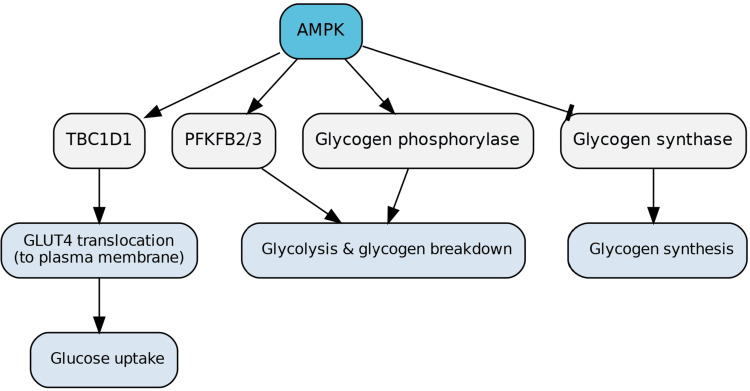
AMPK Regulation of Glucose Uptake and Metabolism in Muscle and Fat In skeletal muscle and adipose tissue, AMPK stimulates glucose uptake by phosphorylating the RabGAP TBC1D1, which promotes GLUT4 translocation to the plasma membrane. AMPK also enhances glycolysis by activating PFKFB2/3, increasing fructose-2,6-bisphosphate levels to accelerate glycolytic flux. Additionally, AMPK promotes glycogen breakdown through activating phosphorylation of glycogen phosphorylase while simultaneously inhibiting glycogen synthesis by phosphorylating glycogen synthase. These combined actions ensure efficient glucose utilization and energy production under metabolic stress. Image credits: Figure created by the author using information from [22,23].

In muscle and fat tissues, AMPK stimulates glucose uptake. It achieves this by phosphorylating the RabGAP (Rab GTPase-activating protein) TBC1D1, which induces the translocation of GLUT4 (not GLUT1, which is more constitutive) to the plasma membrane [22]. AMPK also enhances glycolysis by activating 6-phosphofructo-2-kinase/fructose-2,6-bisphosphatase 2/3 (PFKFB2/3) and promotes glycogen breakdown through the activating phosphorylation of glycogen phosphorylase. Concurrently, it blocks glycogen synthesis by phosphorylating and inhibiting glycogen synthase (Figure 8) [23].

**Figure 9:**
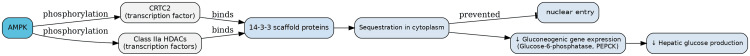
AMPK Regulation of Gluconeogenesis in the Liver AMPK suppresses hepatic glucose production by phosphorylating and inhibiting key transcriptional regulators of gluconeogenesis. In response to glucagon signaling, dephosphorylated CRTC2 and class IIa HDACs translocate to the nucleus, where they bind CREB and FOXO1, respectively, to drive expression of gluconeogenic enzymes such as glucose-6-phosphatase and PEPCK. AMPK phosphorylation of CRTC2 and class IIa HDACs promotes their binding to 14-3-3 scaffold proteins, which sequester them in the cytoplasm and prevent transcriptional activation of gluconeogenesis. Image credits: Figure created by the author using information from [2,23].

In the liver, AMPK effectively downregulates gluconeogenesis by inhibiting key transcription factors such as CREB-regulated transcription coactivator 2 (CRTC2) and class IIa histone deacetylases (HDACs) [23]. In response to glucagon, dephosphorylation of CRTC2 and class IIa HDACs allows them to bind to CREB and FOXO1, respectively, triggering gluconeogenesis by enhancing the expression of glucose-6-phosphatase and phosphoenolpyruvate carboxykinase (PEPCK). However, phosphorylation of class IIa HDACs and CRTC2 by AMPK facilitates the binding of 14-3-3 scaffold proteins, which sequester these transcription factors into the cytoplasm, thereby suppressing hepatic glucose production (Figure 9) [2].

Regulation of mTORC1

The activity of mTORC1 is tightly controlled by changes in a cell's nutrient and environmental status. Because mTORC1's primary functions are anabolic, it's only active when a cell is in an energy-rich environment with ample nutrients, oxygen, and growth factors. When these conditions are met, mTORC1 becomes poised to receive upstream signals from two key small GTPases: Rheb and Rag GTPases [24,25].

To integrate these signals, mTORC1 must first be recruited to the surface of the lysosome, a process mediated by its anchoring to the Rag GTPase complex. This translocation to the lysosomal membrane is a critical step, as it brings mTORC1 into proximity with its activator, Rheb GTPase. This spatial arrangement allows Rheb to directly stimulate the kinase activity of mTORC1, initiating the downstream anabolic cascade [26].

Growth Factors

**Figure 10:**
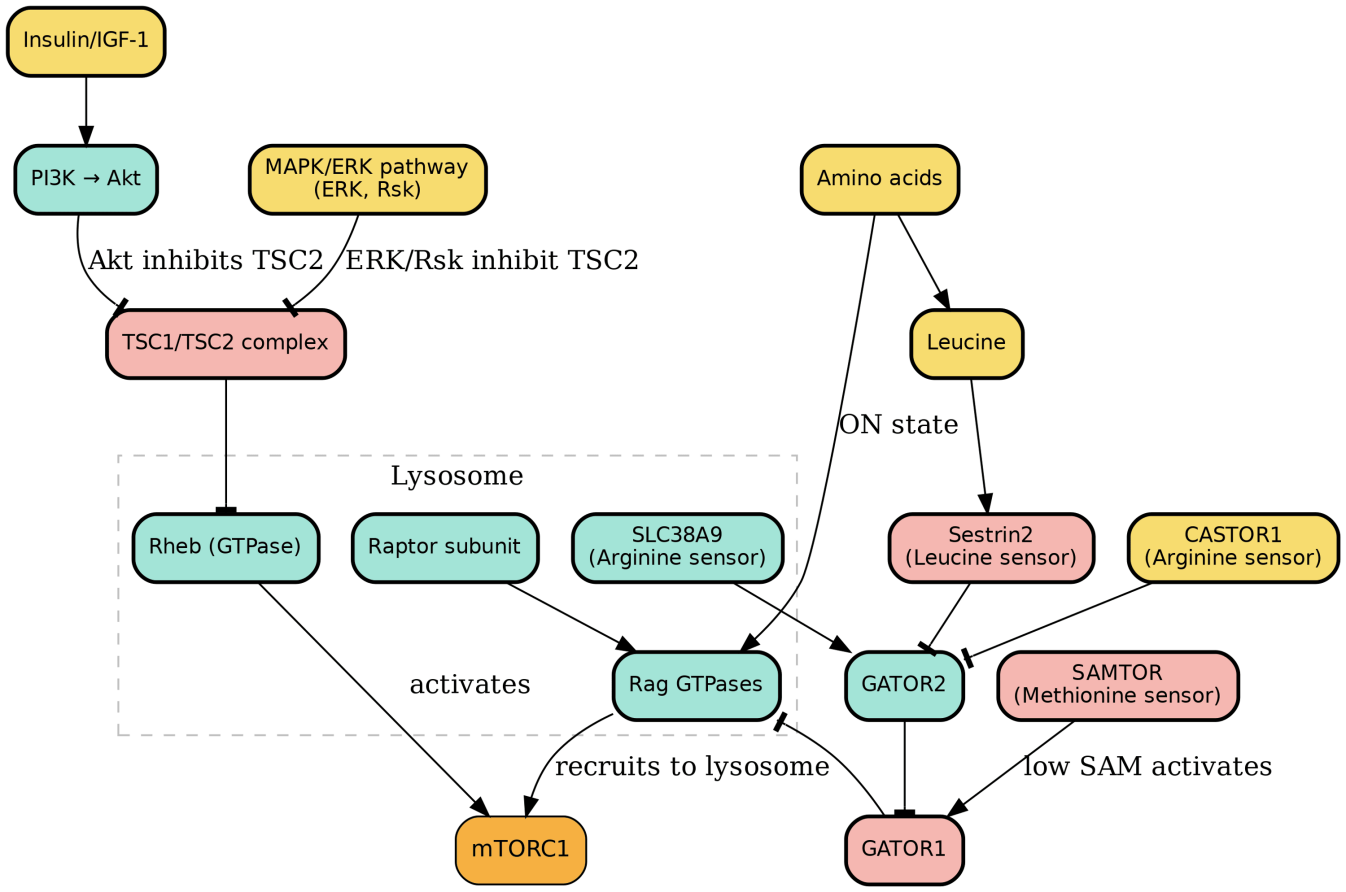
Upstream Regulation of the mTOR Signaling Pathway mTORC1 activity is tightly controlled by upstream nutrient and growth factor signals. Growth factor signaling through the MAPK/ERK cascade leads to ERK- and Rsk-mediated phosphorylation of TSC2, inactivating the TSC1/TSC2 complex and enabling Rheb to remain in its active GTP-bound state, thereby stimulating mTORC1. Insulin and IGF-1 signaling similarly activate mTORC1 through the PI3K/Akt pathway, where Akt phosphorylates TSC2 at multiple residues, disrupting its GAP activity and localization, further sustaining Rheb activation. Amino acids regulate mTORC1 via Rag GTPases, which recruit the mTORC1 complex to the lysosomal surface for activation by Rheb-GTP. Rag activity is coordinated by the GATOR complexes and nutrient sensors: Sestrin2 (leucine sensor), CASTOR1 (arginine sensor), SLC38A9 (lysosomal arginine sensor), and SAMTOR (methionine/SAM sensor). These sensing mechanisms ensure that mTORC1 activation occurs only under nutrient-rich conditions, enabling synergistic integration of growth factor and amino acid signals. Image credits: Figure created by the author using information from [1,27-42].

Growth factors are crucial upstream signals that activate the mTORC1 pathway. TSC acts as a major inhibitor of mTORC1 by converting Rheb from its active, GTP-bound state to its inactive, GDP-bound state. The MAPK/ERK pathway, which is activated by growth factor signaling, inhibits this TSC1/TSC2 complex. When a growth factor binds to its receptor (e.g., EGFR), a cascade is initiated: Grb2 recruits Sos, activating Raf, which in turn activates Mek, followed by ERK, and finally Rsk. Both ERK and Rsk then phosphorylate TSC2, specifically at serine residue 644, deactivating it [27]. This inactivation of TSC2 removes the brake on Rheb, allowing it to remain in its GTP-bound state and promote mTORC1 activation (Figure 10) [1].

Insulin and insulin-like growth factor 1 (IGF-1) are also key upstream activators of mTORC1. Upon insulin binding to its receptor, it activates the PI3K/Akt pathway. This begins with the activation of docking proteins like insulin receptor substrate 1 (IRS-1), which recruits Phosphatidylinositol-3-kinase (PI-3K). PI-3K then converts PIP2 to PIP3, creating a docking site for both PDPK1 and Akt. The activation of Akt is central to this pathway, as it phosphorylates TSC2 at multiple serine and threonine residues, including serine 939, serine 981, and threonine 1462 [28]. This phosphorylation disrupts the TSC1/TSC2 dimer, preventing it from functioning as a Rheb-GAP and from anchoring to the lysosomal surface. As a result, Rheb remains active, and mTORC1 is stimulated (Figure 10) [29,30-33].

Amino Acids

Amino acids are the most critical nutrients for activating mTORC1, and this regulation is dependent on the activity of Rag GTPases. The Rag GTPases are heterodimers composed of RagA/B and RagC/D, which are anchored to the lysosomal surface by the Ragulator complex [34]. In the presence of abundant amino acids, RagA/B is in its active, GTP-bound state, while RagC/D is in its inactive, GDP-bound state. In this "on state," the Raptor subunit of mTORC1 directly binds to the Rag GTPases, recruiting mTORC1 to the lysosomal membrane. This translocation positions mTORC1 to be activated by Rheb-GTP, leading to a powerful synergistic effect on mTORC1 activity (Figure 10) [35-37].

The regulation of Rag GTPase activity is intricate and involves several amino acid-sensing complexes. When cytosolic amino acid concentrations are low, the GATOR1 complex acts as a GTPase-activating protein (GAP) for RagA/B, hydrolyzing its GTP and inactivating the Rag complex, thereby suppressing mTORC1 activity [1]. The GATOR2 complex functions as an antagonist to GATOR1, and its activity is inhibited by various amino acid sensors. For instance, Sestrin2 acts as a leucine sensor; when leucine levels are low, Sestrin2 binds to and inhibits GATOR2, allowing GATOR1 to suppress mTORC1. Conversely, high leucine levels release Sestrin2 from GATOR2, relieving the inhibition on mTORC1 [38]. Similarly, CASTOR1 (cellular arginine sensor for mTORC1) is a cytosolic arginine sensor that inhibits GATOR2 when arginine is scarce [39]. On the other hand, the lysosomal membrane protein SLC38A9 serves as a lysosomal arginine sensor. When it detects arginine, it facilitates the efflux of neutral amino acids, such as leucine, into the cytosol, which in turn promotes GATOR2 activity and upregulates mTORC1 (Figure 10) [40,41].

Methionine also plays a key role in this regulatory network. S-adenosylmethionine (SAM), a metabolic byproduct of methionine, acts as an allosteric regulator. A low concentration of SAM activates the SAM-sensing protein SAMTOR, which then binds to GATOR1 and the KICSTOR complex to inhibit mTORC1. Therefore, an abundant concentration of methionine, and thus SAM, stimulates mTORC1 activity by preventing this inhibition [42].

Regulation of mTORC2

The detailed mechanism of mTORC2 activation is less understood compared to mTORC1, but studies have consistently shown that growth factors and the PI3K pathway play crucial roles in its regulation. A key component of this regulation is the mTORC2 subunit mSin1, which typically autoinhibits mTORC2 kinase activity in the absence of stimuli like IGF-1 or insulin. When the PI3K pathway is activated by these growth factors, the lipid second messenger phosphatidylinositol (3,4,5)-trisphosphate (PIP3) is produced. PIP3 then binds to mTORC2, relieving the autoinhibition by mSin1 and activating the complex at the plasma membrane [43]. mTORC2 and its downstream target Akt can also form a positive feedback loop. Activated Akt can negatively phosphorylate mSin1, further activating mTORC2, which in turn stimulates Akt to upregulate mTORC1 [44]. Recent studies have also shown that Ras can directly stimulate mTORC2 kinase activity, suggesting a pathway that may have significant implications for cancer, cell survival, and proliferation [45]. Paradoxically, the mTORC2 signaling pathway can also be regulated by mTORC1 through a negative feedback loop. Activated mTORC1 promotes the degradation of Insulin Receptor Substrate 1 (IRS-1), thereby disrupting the PI3K-Akt pathway. Additionally, mTORC1 activates Grb10, a negative regulator of the insulin/IGF-1 receptor, further dampening growth factor signaling [46,47].

Regulation of AMPK

AMPK is activated in response to an increase in the cellular AMP/ADP-to-ATP ratio, which occurs during states of energy stress such as starvation, exercise, ischemia, and mitochondrial inhibition. This activation is mediated primarily by upstream kinases that phosphorylate the AMPKα catalytic subunit at threonine-172 (Thr-172). Liver kinase B1 (LKB1) is a key upstream kinase whose activity is promoted by a high AMP/ADP-to-ATP ratio. The binding of AMP or ADP to the AMPK γ-subunit not only enhances LKB1's phosphorylation of Thr-172 but also induces a conformational change in AMPK that protects Thr-172 from dephosphorylation by protein phosphatases. Furthermore, AMP binding allosterically activates AMPK, increasing its activity by up to 10-fold (Figure 6) [48].

**Figure 11:**
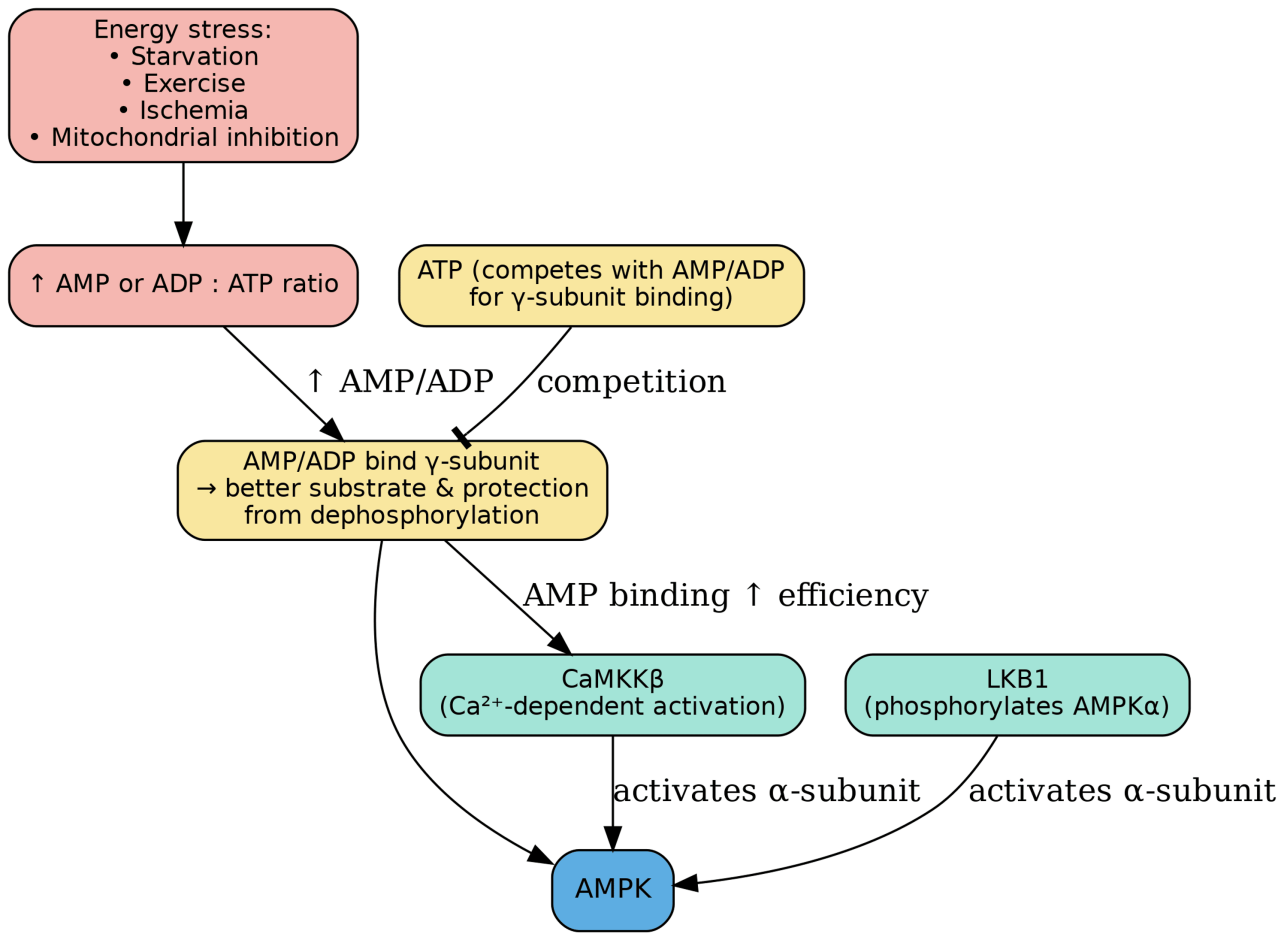
Upstream Regulators of AMPK Activation AMPK is activated by cellular energy stress through changes in the AMP/ADP-to-ATP ratio. Elevated AMP or ADP levels promote phosphorylation of the AMPKα subunit at Thr-172 by LKB1, the primary upstream kinase. Binding of AMP or ADP to the AMPKγ subunit not only enhances LKB1-mediated phosphorylation but also protects Thr-172 from dephosphorylation and provides direct allosteric activation of AMPK, increasing its activity up to 10-fold. AMPK can also be activated independently of nucleotide levels by CaMKKβ, which responds to rises in intracellular calcium. CaMKKβ phosphorylates AMPK at Thr-172, with AMP binding further enhancing substrate recognition. Together, these mechanisms integrate metabolic and calcium signals to fine-tune AMPK activation. Image credits: Figure created by the author using information from [48].

Alternatively, Ca2+/calmodulin-dependent protein kinase β (CaMKKβ) provides a nucleotide-independent mechanism for AMPK activation. CaMKKβ senses intracellular calcium levels rather than the cellular energy status. In response to high calcium concentrations, CaMKKβ directly phosphorylates AMPK at Thr-172, thereby activating it. While CaMKKβ activity itself isn't directly controlled by AMP/ADP/ATP levels, the binding of AMP to the AMPK γ-subunit makes the AMPKα subunit a more favorable substrate for CaMKKβ. This integration means that CaMKKβ, despite its calcium-sensing role, is still an important player in regulating AMPK activity in response to the overall cellular energy state (Figure 11) [48].

Discussion

mTOR and Skeletal Muscle Hypertrophy

**Figure 12:**
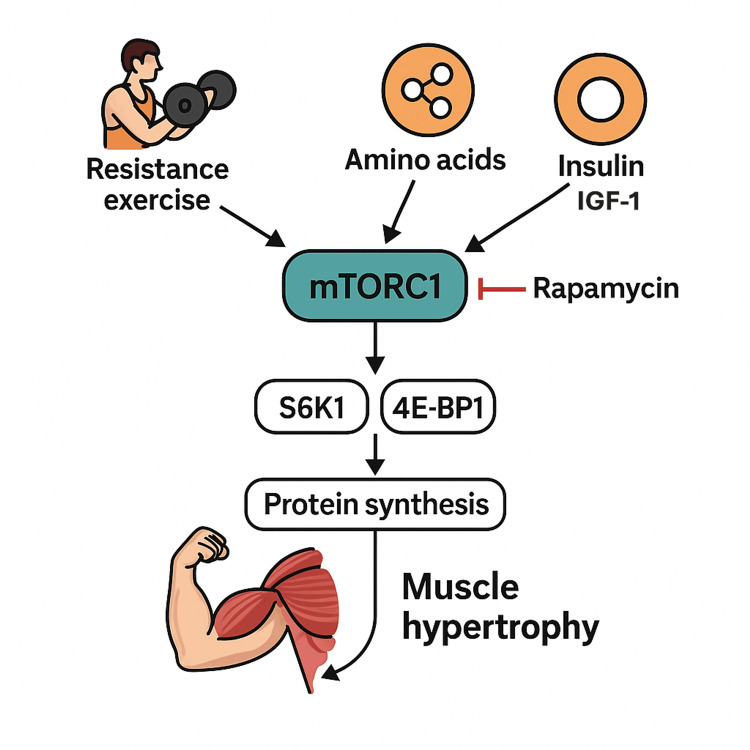
Anabolic Control of Muscle Mass by mTOR Skeletal muscle mass is regulated by the balance between anabolic and catabolic processes, with mTOR acting as a central coordinator of growth. Anabolic signals - including resistance exercise, growth factor stimulation, and adequate nutrient availability - activate the mTOR pathway to promote protein synthesis and muscle hypertrophy. Conversely, insufficient activation of mTOR shifts the balance toward catabolic pathways, leading to muscle wasting. Image credits: Figure created by the author using information from [49-70].

The maintenance of skeletal muscle mass is a delicate balance between anabolic (building) and catabolic (breaking down) processes. The mTOR, a serine/threonine kinase, plays a critical role in controlling this balance. While mTOR's function in promoting protein synthesis is evolutionarily conserved, recent findings have further expanded our understanding of its role in regulating muscle mass [49]. The anabolic signals that drive muscle growth and the catabolic signals that lead to muscle wasting are largely controlled by mTOR. Therefore, for muscle mass to increase, anabolic processes must significantly outweigh catabolic ones. This anabolic shift can be effectively provoked by three key components: resistance exercise, increased growth factors, and adequate nutrient intake, all of which activate the mTOR signaling pathway (Figure 12).

Resistance exercise: Anabolic stimuli, particularly resistance exercise, significantly contribute to the physiological control of the mTOR pathway in human skeletal muscle. Resistance exercise has been shown to acutely increase muscle protein synthesis (MPS) even in a fasted state. This is accompanied by an increase in the phosphorylation of mTOR's key downstream effectors, S6K1 and 4E-BP1, indicating that the pathway is activated for at least 24 hours post-exercise [50]. A strong correlation between the magnitude of post-exercise S6K1 phosphorylation and the degree of hypertrophy following a period of resistance training has been observed in several human studies. This evidence suggests that resistance exercise-induced hypertrophy is mediated by a rapamycin-sensitive, mTOR/mTORC1-dependent mechanism, similar to hypertrophy from chronic mechanical overload [51].

Initial evidence supporting this hypothesis emerged from animal studies. In 1999, Baar and Esser demonstrated that a single bout of resistance exercise-like contractions in rats led to a dose-dependent increase in S6K1 phosphorylation, and this increase was closely linked to the induction of hypertrophy. Since changes in S6K1 phosphorylation are primarily regulated by a rapamycin-sensitive pathway, this work was among the first to suggest a role for mTOR-dependent signaling in hypertrophy [52]. This was further substantiated in 2001 by Bodine et al., who found that systemic administration of rapamycin, an mTORC1 inhibitor, prevented the increase in S6K1 phosphorylation and, more critically, completely blocked the hypertrophic response following chronic mechanical overload via synergist ablation [53]. These findings were subsequently replicated by other research groups, solidifying the notion that mTORC1 activity is essential for skeletal muscle hypertrophy.

Human studies have since corroborated these findings. In 2009, Drummond et al. found that rapamycin treatment inhibited the early (one to two hours) acute contraction-induced increase in protein synthesis in humans by 40%. The study also revealed that rapamycin prevented the typical exercise-induced phosphorylation of S6K1, while phosphorylation of eEF2 was unaffected, indicating that translation elongation was not inhibited. Interestingly, rapamycin administration before exercise also prevented the contraction-induced increase in phosphorylation of ERK1/2 and attenuated the increase in MNK1 phosphorylation. However, phosphorylation of eIF4E, a known MNK1 target, remained unchanged, suggesting that rapamycin does not directly suppress MAPK signaling. The authors concluded that mTORC1 signaling is critical for regulating contraction-induced MPS, and that coordinated activation of both mTORC1 and ERK1/2 may be necessary for maximal protein synthesis in human skeletal muscle [54].

An important early human study in 2008 by Terzis et al. further investigated the long-term link between S6K1 phosphorylation and hypertrophy. Eight male volunteers underwent a single session of resistance training (leg press) and a 14-week resistance training program. Muscle biopsies from the vastus lateralis showed that the increase in S6K1 phosphorylation at Thr389 30 minutes after the initial session was strongly correlated with the long-term percentage increases in fat-free mass (r = 0.89), fat-free mass of the legs (r = 0.81), and type IIA muscle fiber cross-sectional area (r = 0.82) after the 14-week training period. These results provided compelling evidence that S6K1 phosphorylation serves as a proxy for the signaling pathways that drive long-term protein accumulation and hypertrophy in human skeletal muscle [55].

Amino acids: Dietary protein and amino acid consumption are powerful stimulators of MPS. The anabolic response is mediated by increased phosphorylation of proteins within the mTORC1 pathway, including S6K1, with the magnitude of this phosphorylation often predicting the extent of skeletal muscle hypertrophy and strength gains. This effect is synergistic, as the combination of resistance exercise and protein or amino acid intake potentiates mTORC1 signaling and protein synthesis in muscle cells [56].

Numerous studies have provided evidence for this synergistic effect. For example, a 2019 study by Edman et al. investigated how protein intake after resistance training affects the mTOR pathway. They found that providing essential amino acids (EAAs) to strength-trained men during and after leg press exercise led to a significant two- and six-fold increase in mTOR and S6K1 phosphorylation, respectively, in both type I and type II muscle fibers compared to a placebo. This demonstrates that EAA supplementation amplifies the anabolic signaling initiated by resistance training [56].

While all amino acids are necessary for building new proteins, leucine is particularly important for activating mTORC1. Leucine, a branched-chain amino acid (BCAA), acts as a key signaling molecule. Muscle cells possess a mechanism that senses intracellular leucine levels, which then activates the Rag GTPase proteins. This activation leads to the translocation of mTOR to the lysosomal surface, where it can be activated by Rheb. Consequently, a high concentration of leucine stimulates the Rag proteins, bringing mTOR and Rheb together to boost protein synthesis and promote muscle hypertrophy [52].

This leucine-driven activation is also dependent on the efficient transport of amino acids into the cell. Intracellular amino acid levels are maintained by a balance between dietary supply and the rate of transport across the cell membrane. The two primary systems that influence mTORC1 signaling are the System L and System A transporters. The System L transporter (LAT1), linked to the glycoprotein CD98, functions as an intracellular amino acid exchanger, importing BCAAs like leucine in exchange for other intracellular amino acids, such as glutamine. The System A transporter, specifically SNAT2, is crucial for regulating intracellular glutamine levels. The cell uses a coordinated process involving these transporters: the Na+/K+ pump maintains an electrochemical gradient, which allows SNAT2 to import glutamine. The resulting increase in intracellular glutamine then drives the exchange of glutamine for extracellular leucine via LAT1. Therefore, the efficiency of this transport process is critical for leucine-induced mTORC1 activation [57].

In addition to protein and amino acids, carbohydrates and insulin also play a key role. A 1999 study by Tipton et al. proposed that an optimal anabolic stimulus involves a combination of carbohydrates to spike insulin and a minimal amount of EAAs to provide building blocks for protein synthesis [58]. This was supported by a 2017 study by Song et al., which demonstrated that a post-workout drink containing both 20 g of protein and 44 g of carbohydrate significantly boosted protein synthesis. The carbohydrate component triggered a substantial insulin response, which enhanced mTOR signaling. This was evidenced by a 10-fold increase in S6K1 activity and a 6-fold increase in Akt activity, along with the exercise and nutrition-induced dissociation of TSC2 from Rheb and the co-localization of mTOR and Rheb [50]. These findings collectively highlight the importance of synergistic signaling from both amino acids and insulin for maximal mTORC1 activation and muscle anabolism after resistance exercise.

Insulin: Insulin's role in promoting skeletal MPS is well-documented in humans [59,60]. At the cellular level, insulin increases the phosphorylation of Akt and mTOR, which in turn boosts the phosphorylation of their downstream targets, 4E-BP1 and S6K1. This enhanced mTORC1 signaling ultimately promotes translation initiation and accelerates MPS [61].

Beyond its direct signaling effects, insulin also stimulates MPS indirectly by increasing muscle blood flow. It activates endothelial nitric oxide synthase (eNOS), which triggers vasodilation and increases capillary recruitment, microvascular volume, and nutritive blood flow to skeletal muscle [62,63]. This improved muscle perfusion exposes more tissue to insulin and a greater supply of amino acids and other nutrients, further stimulating MPS through the Akt/mTORC1 pathway [64].

However, insulin's influence on adult human muscle is complex and can be affected by factors like amino acid availability, muscle blood flow, and endothelial health [65]. While some studies show that hyperinsulinemia alone has no significant effect on MPS, they often report a notable reduction in muscle protein breakdown (proteolysis) [66].

This anabolic effect of insulin is also diminished with age. In healthy, non-diabetic elderly individuals, skeletal muscle is resistant to insulin's anabolic effects, a condition linked to reduced vasodilation and impaired Akt/mTORC1 signaling [67]. A 2006 study by Rasmussen et al. demonstrated that, unlike in younger subjects, exposing older muscle to local hyperinsulinemia did not increase MPS. The study found a strong correlation between changes in muscle blood flow and amino acid availability, but no link between amino acid concentration and protein synthesis. This suggests that a diminished vasodilatory response to insulin in older muscle may be a key factor in its reduced anabolic effect, as it impairs nutrient delivery [67].

A 2016 meta-analysis by Abdulla et al. summarized these findings, concluding that insulin's role in skeletal muscle anabolism is critical but highly dependent on the rate of amino acid delivery. They noted that supraphysiological concentrations of insulin are often required to stimulate anabolism in the absence of amino acids. However, the most consistent effect of insulin is to reduce muscle protein breakdown, an effect that is compromised in older adults and those with insulin resistance. This resistance is likely due to impaired insulin signaling in muscle protein metabolism and endothelial dysfunction rather than poor glucose tolerance [68].

IGF-1: IGF-1 is a well-studied growth factor that is crucial for regulating muscle size and function. Many of the beneficial effects of physical activity are thought to be mediated by IGF-1 signaling [69]. IGF-1 stimulates protein synthesis in skeletal muscle primarily through two major pathways: the PI3K/Akt/mTOR pathway and the PI3K/Akt/GSK3 pathway [70]. The IGF-1/Akt/mTOR pathway is particularly essential for promoting muscle growth [71].

Within this pathway, Akt phosphorylates and inhibits the TSC1/TSC2. This inhibition allows Rheb, a small G-protein, to remain in its active, GTP-bound state. Active Rheb then directly stimulates mTORC1, which in turn phosphorylates key downstream targets. Specifically, mTORC1 phosphorylates S6K1, which enhances protein synthesis by activating ribosomal protein S6, a component of the 40S ribosomal subunit. Additionally, mTORC1 phosphorylates 4E-BP1, causing it to dissociate from its inhibitory complex with the translation initiation factor eIF4E. This release allows eIF4E to bind to eIF4G, forming the essential translation initiation complex and promoting protein synthesis [72]. The critical role of this pathway was demonstrated in animal studies where administration of the mTOR inhibitor rapamycin blocked S6K1 phosphorylation, prevented the release of 4E-BP1, and ultimately inhibited surgically induced muscle hypertrophy [53]. Consistent with these findings, the Akt/mTOR pathway is suppressed during disuse-induced muscle atrophy and is reactivated upon reloading.

Beyond the mTOR pathway, Akt-mediated activation of glycogen synthase kinase-3 (GSK3) is another important downstream effector of IGF-1 signaling. In hypertrophic muscle, GSK3 is phosphorylated and its activity is reduced, which leads to the activation of eukaryotic translation initiation factor 2B (eIF2B) and the transcriptional co-activator β-catenin [73,74]. IGF-1 also plays a role in skeletal muscle regeneration by activating muscle stem (satellite) cells, which contribute to hypertrophy and prevent atrophy. Conversely, in many chronic illness states, IGF-1 levels and downstream signaling are inhibited, leading to muscle atrophy through a combination of reduced protein synthesis, increased activity of the ubiquitin-proteasome system (UPS), enhanced autophagy, and impaired muscle regeneration [70].

AMPK Activation in Skeletal Muscle Cells

Skeletal muscle is highly adaptable to various physiological demands. AMPK functions as an intracellular energy sensor, regulating both anabolic and catabolic pathways to maintain cellular energy homeostasis. This role is particularly critical in tissues with high energy turnover, such as muscle. When muscle ATP consumption increases during exercise, the adenylate kinase reaction can rapidly increase intracellular AMP concentrations. This elevates the cellular AMP/ATP and ADP/ATP ratios, which serve as a powerful signal to activate AMPK [75].

Acute pharmacological activation of AMPK promotes glucose transport and fatty acid oxidation in skeletal muscle while simultaneously inhibiting glycogen synthase activity and protein synthesis [76,77]. During muscle contraction, the cellular energy charge decreases in a manner dependent on the exercise duration and intensity [78]. The resulting increase in intracellular AMP/ATP and ADP/ATP ratios activates AMPK [75]. The binding of AMP or ADP to the γ-subunit's Bateman domains induces a conformational change that provides up to a 10-fold allosteric activation [79]. More importantly, this conformational change enhances the phosphorylation of the α-subunit at threonine-172 (Thr-172) by upstream kinases [80,81] and protects it from dephosphorylation by protein phosphatases like PP1, PP2A, and PP2C [82]. The combined effect of allosteric activation and Thr-172 phosphorylation can increase AMPK activity by over 1000-fold [79].

**Figure 13:**
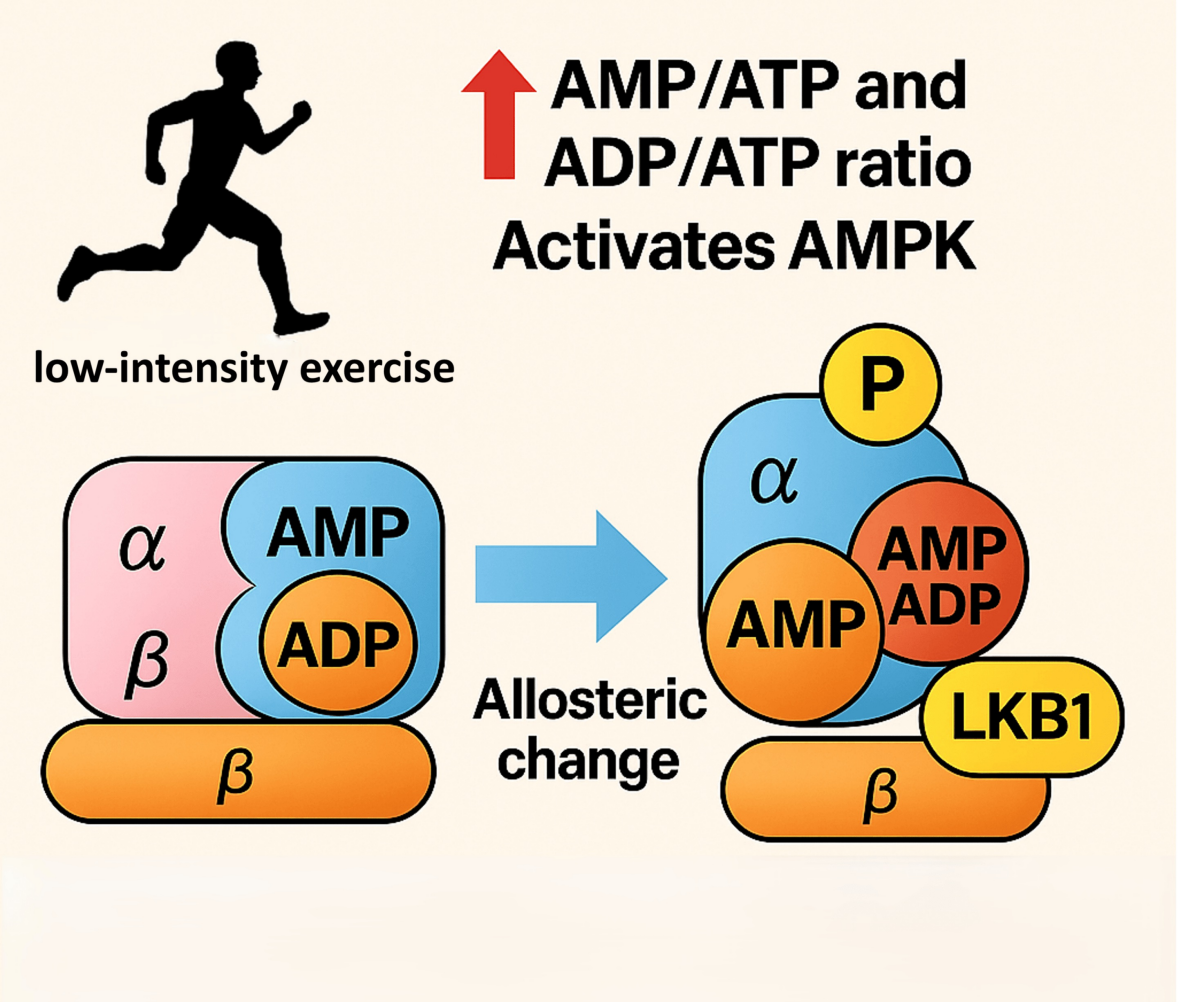
Exercise-Dependent Activation of AMPK Complexes Different upstream kinases regulate AMPK depending on the exercise stimulus: LKB1 predominantly activates α2-containing AMPK complexes during high-intensity or pharmacological stress, while CaMKKβ activates α1-containing complexes during prolonged, low-intensity exercise through calcium signaling. Glycogen further modulates AMPK activity via its glycogen-binding domain, which inhibits α2-containing complexes when bound. The overall AMPK response is thus tailored to exercise intensity and duration, fine-tuning skeletal muscle adaptation. Image credits: Figure created by the author using information from [75-95].

AMPK is activated by different upstream kinases depending on the exercise stimulus. LKB1 is considered the primary upstream kinase in skeletal muscle, responsible for phosphorylating α2-containing AMPK complexes in response to muscle contraction and pharmacological activators [83,84]. In contrast, during prolonged, low-intensity exercise, the phosphorylation and activation of α1-containing AMPK complexes are triggered by Ca2+/calmodulin-dependent protein kinase kinase β (CaMKKβ) [85,86]. While LKB1 is constitutively active [87,88], CaMKKβ activates AMPK when intracellular Ca2+ concentrations rise, even in the absence of an imbalance in adenine nucleotide content [89]. Furthermore, glycogen has been shown to modulate AMPK activity through its interaction with the β-subunit. The β-subunit contains a glycogen-binding domain (GBD) that causes AMPK complexes to associate with glycogen particles, and this interaction has been found to inhibit AMPK function, particularly for the α2-containing complexes (Figure 13) [90,91].

The activation profile of AMPK is sensitive to exercise intensity and duration. AMPK activation is typically detected at exercise intensities of at least 60% of VO2peak [92,93], but can also be activated by low-intensity exercise (e.g., 30-40% of VO2peak) performed to exhaustion [94]. The specific AMPK heterotrimers are regulated differently based on the stimulus. During short-duration, high-intensity exercise, LKB1 is the primary kinase activating α2-containing complexes. In contrast, prolonged, low-intensity exercise predominantly activates and phosphorylates α1-containing complexes via CaMKKβ. As a result, the specific functional response to muscle contraction is dictated by which AMPK complex is activated, an outcome determined by the intensity and duration of the exercise (Figure 13) [95].

AMPK and Skeletal Muscle Hypertrophy

Given its role in catabolic and anti-anabolic processes, AMPK activity has long been hypothesized to inhibit muscle development. Current evidence strongly supports this notion of a transient inhibition [96]. Studies comparing the hypertrophic response of rat muscle to synergist ablation-induced loading have shown that high AMPK phosphorylation is associated with reduced muscle hypertrophy and impaired activation of the mTOR pathway [97].

Direct pharmacological evidence further demonstrates that AMPK activation reduces muscle growth. The AMP analog 5-aminoimidazole-4-carboxamide ribonucleotide (AICAR), a potent AMPK activator, was shown to significantly reduce the mTOR signaling response when injected an hour before a bout of resistance exercise [98]. This suggests that AMPK activation inhibits the normal increase in protein translation that follows resistance exercise. Similarly, continuous perfusion of AICAR to overloaded plantaris muscles substantially reduced muscular growth following synergist ablation [99].

Genetic evidence from Mounier et al. further supports AMPK's inhibitory role in skeletal muscle growth. Using synergist ablations on AMPKα1 knockout mice, the researchers found that despite having lower basal muscle mass, the knockout mice exhibited superior whole-muscle growth and fiber hypertrophy 7 and 21 days after overload. The AMPKα1-knockout muscles showed higher activation of the mTOR pathway (as indicated by S6K1 and 4E-BP1 phosphorylation) and reduced eEF2 phosphorylation (reflecting enhanced eEF2 activity). This occurred despite a compensatory increase in AMPKα2 activity in the knockout muscles, suggesting that the AMPKα1 isoform is the primary regulator of overload-induced muscle growth [100].

The evidence for AMPK's anti-anabolic effects raises the question of whether its activation during exercise inhibits a muscle's ability to hypertrophy. Atherton et al. [101] demonstrated that different exercise modalities elicit distinct signaling responses in rat skeletal muscle. An in vitro electrical stimulation protocol that mimicked resistance exercise (high-frequency, intermittent) increased phosphorylation of Akt, TSC2, mTOR, and its downstream targets, along with a rise in protein synthesis. In contrast, endurance-type stimulation (low-frequency, continuous) increased AMPK activation and the accumulation of peroxisome proliferator-activated receptor γ coactivator-1α (PGC-1α) [101].

Recent evidence suggests that concurrent endurance and resistance training may produce modest interference effects on lower-body strength in males, though the impact on hypertrophy and overall strength is nuanced and depends on factors such as training status, sex, and the frequency and duration of endurance exercise [102]. A meta-analysis by Wilson et al. showed a negative effect of endurance exercise on muscular hypertrophy, strength, and power, which is dependent on the frequency and duration of the endurance training [103]. However, the specific molecular mechanisms underlying this interference effect in humans are less clear [104]. For instance, a study by Apró et al. found that activating AMPKα2 via one hour of vigorous cycling did not affect the subsequent activation of the mTOR pathway or mixed MPS following a resistance training bout in trained men [105]. The situation is further complicated by the fact that while acute AMPK activation is inhibited in endurance-trained muscle immediately after exercise [106], chronic endurance training increases both the protein level and basal activity of the AMPKα1 isoform (Table 1). Consequently, whether AMPKα1 activation from endurance training directly interferes with the anabolic signaling and hypertrophy from resistance exercise remains an open question [96].

**Table 1:** Principal Human Studies of Concurrent (Endurance + Resistance) Training Key human trials and reviews exploring how endurance and resistance exercise interact at the molecular and performance levels. Early meta-analyses confirmed that concurrent training can attenuate hypertrophy and strength gains, primarily when endurance sessions are frequent, long, or poorly sequenced relative to resistance training. More recent mechanistic studies show this effect is context-dependent: acute AMPK activation from endurance exercise does not necessarily inhibit mTORC1 or S6K1 activity if sufficient recovery time is allowed. Sex, training status, and session order influence the magnitude of the “interference effect.”

Study	Population	Intervention	Duration	Main Findings	Relevance
Atherton et al., 2005 [101]	Healthy adults, electrically stimulated muscle	Endurance-like vs. resistance-like electrical stimulation	Acute	Endurance-like pattern → selective AMPK-PGC-1α activation; resistance-like pattern → PKB-TSC2-mTOR activation. No overlap in signal dominance.	Provides direct molecular explanation for endurance/resistance specificity in adaptation.
Huiberts et al., 2024 [102]	Systematic review and meta-analysis (46 trials; >1,000 participants)	Concurrent training (various)	4-24 weeks	Hypertrophy effect size -0.23 overall. Men showed less interference (-0.15) vs. women (-0.31). Trained participants more resistant to interference.	Sex and training status influence the interference magnitude.
Wilson et al., 2012 [103]	Meta-analysis (21 studies; 422 subjects)	Concurrent endurance + RT vs. RT alone	6-24 weeks	Strength gains reduced by ~31%, hypertrophy by ~12%, power by ~18% vs. RT alone (p < 0.05). Effect greater when endurance frequency ≥ 3×/week or duration > 40 minute. Running caused more interference than cycling.	Quantifies dose- and modality-dependent interference; manage endurance volume.
Coffey and Hawley, 2017 [104]	Mechanistic review of human trials	Endurance + resistance sequencing	-	Summarized multiple studies: performing endurance → RT within < 3 hour blunted mTOR-S6K1 signaling ~ 40-60%; spacing ≥ 6 hour preserved activation. Mitochondrial biogenesis (PGC-1α) unaffected by RT when sessions separated.	Timing and recovery between modalities are major determinants of molecular interference.
Apró et al., 2015 [105]	9 trained men	10 × 60 seconds cycling intervals (90% VO₂max) → 15 min rest → leg press RE	Acute	AMPK Thr172 phosphorylation ↑ ~three-fold after HIIT, but S6K1 phosphorylation ↑ ~3.5-fold post-RE (p < 0.01); mTORC1 activity intact.	High-intensity cycling did not inhibit RE-induced anabolic signaling, suggesting tolerance in trained muscle.
McConell et al., 2005 [106]	7 active men	10 days of endurance training (cycling, 65% VO₂max, 90 min/day)	90 minutes bout pre-/post-training	AMPK phosphorylation during prolonged exercise ↓ 45% post-training (p < 0.05) despite similar glycogen levels; ACC Ser79 ↓ 48%.	Endurance training reduces AMPK activation over time, mitigating interference potential chronically.

mTOR and Diseases

Appropriate control of mTOR is essential for maintaining homeostasis and overall health. However, abnormalities in mTOR activity can lead to metabolic dysregulation and disease. Overfeeding, in particular, readily hyperactivates the mTOR pathway, which is a key regulator of glucose metabolism and lipogenesis in various tissues. This hyperactivation is believed to be a contributing factor in many diseases of constitutive growth, such as obesity and type 2 diabetes [1].

Metabolic syndrome: Dysfunction in mTOR signaling is strongly linked to the symptoms of type 2 diabetes, including whole-body insulin resistance, hyperglycemia, and hyperlipidemia. A key mechanism behind insulin resistance is the mTORC1-mediated negative feedback loop. This loop, which is driven by mTORC1 activating S6K1 and Grb10, suppresses downstream PI3K pathway effectors. The critical role of this feedback loop is highlighted by studies showing that S6K1-deficient animals are protected from high-fat diet-induced insulin resistance [107]. Excessive nutrients and mitogens can also disrupt PI3K-mTORC2 signaling, leading to insulin resistance and the buildup of ectopic lipids in muscle and liver, which are hallmarks of type 2 diabetes. Because long-term rapamycin treatment also inhibits mTORC2, rapamycin-based therapies have been unsuccessful for diabetic patients with hyperactive mTORC1 signaling [1].

The liver's role in controlling whole-body metabolism is particularly significant. In a healthy state, insulin stimulates lipogenesis and inhibits gluconeogenesis. However, in type 2 diabetes, a signaling bifurcation upstream of mTORC1 allows lipogenesis to continue while insulin fails to inhibit gluconeogenesis [108]. This occurs because defective Akt signaling fails to inhibit the transcription factor FoxO, which increases the expression of gluconeogenic genes, while mTORC1 maintains lipogenesis via the transcription factor SREBP1. This selective insulin resistance promotes hepatic glucose production and triglyceride accumulation, resulting in hyperglycemia and hyperlipidemia. While this model has been widely accepted, recent studies have raised questions about its simplicity, suggesting that mTORC1 is required, but not sufficient, for hepatic lipogenesis [109].

The complexity of metabolic illnesses like type 2 diabetes is further highlighted by the various co-morbidities that are either caused by or contribute to insulin resistance. These include hepatic steatosis, obesity, metabolic syndrome, and diabetic nephropathy. Hepatic steatosis, or fatty liver disease, is the abnormal accumulation of triglycerides (TGs) in liver cells, which occurs because insulin resistance causes the liver to increase both the production and uptake of fatty acids, resulting in the excessive storage of TGs as lipid droplets [110]. It is facilitated by mTORC1's ability to be activated by nutrients even in the absence of insulin signaling [111]. Diabetic nephropathy, a kidney complication of diabetes, is induced by chronic high blood glucose levels. Recent independent studies have revealed a role for mTOR signaling in podocyte function and diabetic nephropathy, suggesting that inhibiting mTOR could offer therapeutic benefits [112].

Therapeutic approaches for type 2 diabetes often target the mTOR pathway. For example, metformin, a first-line treatment, potently inhibits mTORC1 by activating AMPK and TSC [113]. Similarly, inactivating the mTORC1 effector S6K1 can protect against diet-induced obesity and improve insulin sensitivity [114]. Unfortunately, direct pharmacological suppression of mTORC1 has produced more complex results. Rapamycin therapy, for instance, can lead to more severe insulin resistance, likely because it also impairs the stability of the mTORC2 complex, thereby dampening the Akt-dependent insulin response [115]. To avoid these side effects, the development of novel, truly selective mTORC1 inhibitors and tissue-specific modulators of mTORC1 action is necessary [1].

Neurodegenerative diseases: mTOR signaling is crucial for protein homeostasis in the brain, balancing synthesis and autophagic breakdown. mTORC1 is known to enhance learning and memory by promoting protein synthesis and strengthening synapses [116]. For instance, mice with a deficiency in 4E-BP1, a key mTORC1 target, exhibit problems with learning, memory, and social behavior [117]. However, excessive mTOR signaling can lead to maladaptive learning, which is a key feature of addiction. A study on alcohol misuse showed that alcohol activates mTOR in the nucleus accumbens, a brain region associated with reward and addiction. A single dose of rapamycin, an mTOR inhibitor, was able to prevent binge drinking in mice [118]. Conversely, some fast-acting antidepressants, like ketamine, appear to exert their effects by activating mTOR and promoting synapse formation [119].

Disruptions in mTOR signaling are linked to various neurological disorders. Patients with TSC, a genetic disease characterized by benign brain tumors, often suffer from intellectual disability, autism, and epilepsy [120]. Mouse models of epilepsy that lack either PTEN or TSC1 show that hyperactive neuronal mTOR is associated with the epileptic phenotype [121,122]. Similarly, the intellectual impairment seen in these patients is likely connected to mTOR's role in learning and memory formation [123]. In fact, in TSC2-deficient mice, overactive mTOR leads to a learning deficit rather than a benefit, likely due to improper storage of unprocessed information [124,125].

The role of mTOR in promoting protein synthesis is also crucial for controlling axon growth, with the PTEN protein acting as a significant inhibitor of axon regeneration [126]. Furthermore, the dual role of mTOR signaling in regulating both protein synthesis and autophagy is deeply connected to various neurodegenerative disorders. Diseases such as Alzheimer's, Parkinson's, and Huntington's are characterized by the abnormal accumulation of misfolded proteins, which leads to neuronal death and symptoms like involuntary tremors, dementia, and memory loss [127]. Research has shown that rapamycin, by inhibiting mTOR, reduces pathology and increases the autophagic clearance of harmful proteins in mouse models of Alzheimer's and Huntington's disease, thereby highlighting a potential, though unproven, therapeutic avenue that warrants further investigation [128].

Cancer: Although the mTOR kinase itself is rarely mutated in cancer, its signaling is frequently disrupted by upstream oncogenic pathways such as PI3K-Akt and Ras-driven MAPK cascades. This results in excessive mTOR signaling in up to 80% of human cancers, making it a critical regulator of cancer cell proliferation, metabolism, and survival [129].

The hyperactivation of mTOR signaling can originate from mutations in both cell surface receptors and intracellular signaling components. Oncogenic mutations in receptor tyrosine kinases (RTKs), for example, lead to ligand-independent activation that drives mTOR-dependent cell growth [130]. Common examples include mutations or amplifications of EGFR, which are found in glioblastomas [131], non-small cell lung cancer (NSCLC) [132], and various other malignancies such as breast, ovarian, and gastric cancers [133].

Intracellular signaling molecules upstream of mTOR, such as PI3K, PTEN, Akt, Ras, and Raf, are frequently mutated across many cancer types. PIK3CA mutations, affecting the catalytic subunit of PI3K, are among the most common in human malignancies [134]. These alterations are observed in breast [135], endometrial [136], and head and neck squamous cell carcinoma (HNSCC) [137], as well as in colorectal, prostate, gastric, and hepatocellular carcinomas [138-140]. The tumor suppressor PTEN is another key target, with frequent inactivation in glioblastomas [141], endometrial cancer [142], melanoma [143], and central nervous system, prostate, and breast cancers [134]. Likewise, mutations in Akt, Ras, and Raf occur across many malignancies, including pancreatic, ovarian, and breast cancers [138,144,145]. Notably, Ras mutations are found in nearly 90% of pancreatic tumors [145,146].

The PI3K/Akt/mTOR (PAM) signaling network integrates extracellular growth signals to regulate cell growth, metabolism, and survival. Activation of RTKs stimulates PI3K, generating phosphatidylinositol (3,4,5)-trisphosphate (PIP3), which recruits Akt to the plasma membrane. Activated Akt phosphorylates and inhibits TSC2, relieving its suppression of Rheb, a GTPase that directly activates mTORC1. Dysregulation of this pathway leads to uncontrolled anabolic signaling and is a hallmark of many cancers [129,134].

In hematologic malignancies such as leukemias, lymphomas, and myelomas, aberrant activation of the PAM pathway results in persistent mTORC1 signaling. In chronic myeloid leukemia (CML), the BCR-Abl fusion protein constitutively stimulates the PI3K/Akt axis, leading to continuous mTOR activation and uncontrolled myeloid proliferation [147]. In acute myeloid leukemia (AML), activating mutations in RTKs such as FLT3 or c-Kit drive sustained PAM signaling, confirmed by hyperphosphorylation of mTORC1 substrates S6K and 4E-BP1 in primary AML samples [148,149]. In mantle cell lymphoma (MCL), chromosomal rearrangements cause Cyclin D1 overexpression, enhancing mTORC1-dependent cell cycle progression [150,151]. Encouragingly, mTOR inhibitors, used alone or in combination with agents such as doxorubicin, have shown therapeutic benefit in MCL and other hematologic cancers [152].

AMPK, IF, and health benefits: IF has emerged as a popular health trend and a powerful dietary intervention. These eating patterns involve restricting food intake for specific periods, typically ranging from 12 to 40 hours [153]. Prolonged fasting induces a metabolic switch in the body, activating AMPK and shifting the cellular state from anabolic to catabolic [154]. This activation, in turn, upregulates genes that promote mitochondrial biogenesis, autophagy, and cellular stress resistance, while simultaneously repressing mTOR signaling and overall protein synthesis [155].

Recent studies suggest that IF regimens can reduce total and visceral fat mass, factors closely linked to an increased risk of diabetes, although these effects are often comparable to those achieved with continuous calorie restriction [156]. During a prolonged caloric deficit, the body's glycogen stores are depleted, leading to rapid lipolysis in adipose tissue and the release of fatty acids and glycerol into the bloodstream [157,158]. This metabolic shift typically occurs 12 to 36 hours after the last meal. The released free fatty acids (FFAs) are transported to the liver, where they undergo β-oxidation to produce ketone bodies, such as β-hydroxybutyrate (β-OHB) and acetoacetate, which can also trigger mitochondrial biogenesis [159]. AMPK's role in this process is crucial; it downregulates lipid synthesis by inhibiting ACC (acetyl-CoA carboxylase) and simultaneously promotes β-oxidation [160].

IF regimens have been shown to significantly reduce insulin resistance, lower fasting glucose levels by 3-6% in individuals with prediabetes, and improve cardiovascular risk factors, including reductions in total cholesterol (6-21%), LDL cholesterol (7-32%), triglycerides (16-42%), and both systolic (3-8%) and diastolic (6-10%) blood pressure after 6 to 24 weeks of intervention [155,161-166]. Consistent with these findings, Hoddy et al. demonstrated that alternate-day fasting in obese adults resulted in comparable reductions in body weight and fat mass regardless of meal timing, along with favorable changes in LDL particle size and a modest decrease in systolic blood pressure, further supporting the cardiometabolic benefits of IF [167].

The balance of AMPK signaling plays a crucial role in maintaining neuronal health, and its dysregulation has been implicated in various neurological diseases. Fasting periods have been shown to protect neurons from dysfunction and degeneration in animal models of neurological disorders, including epilepsy, Alzheimer’s disease [168], Parkinson’s disease [169], and stroke [170]. While human studies on IF and cognitive performance are limited, some have reported improvements in cognitive domains such as executive function during prolonged fasting [171]. In terms of physical performance, a recent randomized controlled study on males found that a month of IF (20 hours of fasting four days a week) combined with resistance exercise enhanced both upper and lower body endurance compared to controls (Table 2) [172].

**Table 2:** Principal Human Trials on Intermittent Fasting, Time-Restricted Feeding, and Alternate-Day Fasting Representative randomized and observational human studies examining metabolic, compositional, and cardiovascular outcomes of intermittent fasting (IF), time-restricted feeding (TRF), and alternate-day fasting (ADF). Collectively, these trials demonstrate comparable weight loss (≈ 4-8%) to continuous calorie restriction (CR), preservation of lean mass when combined with resistance training, improved insulin sensitivity, lipid and glycemic control, and favorable cardiometabolic risk profiles. Benefits are highly dependent on adherence, feeding window timing, and duration (> 8 weeks yields more consistent effects).

Study	Population	Intervention	Duration	Main Findings	Relevance
Rynders et al., 2019 [153]	50 human trials review	IF/TRF vs. CER	-	Average weight loss 4-10%; FPG -5-10 mg/dL; TG -15-20 mg/dL; adherence > 70%.	Synthesis showing equal or greater metabolic benefit vs. CER.
Khalafi et al., 2024 [156]	84 RCTs (> 3,000 adults)	IF ≥ 8 weeks	Meta-analysis	Pooled weight -3.9 kg; BMI -1.2 kg/m²; waist -3.2 cm; FPG -8 mg/dL; TG -18 mg/dL; LDL -9 mg/dL (p < 0.001).	Confirms robust medium-term cardiometabolic improvement.
Horne et al., 2012 [160]	4,629 patients undergoing angiography	Routine periodic fasting (24 hour monthly) vs. non-fasters	Cross-sectional	Fasting associated with 45% lower odds of diabetes (OR 0.55, 95% CI 0.36-0.84) and 40% lower CAD (OR 0.60).	Observational support linking habitual fasting with lower cardiometabolic risk.
Trepanowski et al., 2017 [161]	100 overweight/obese adults (BMI ≈ 34)	ADF: 25% of needs (≈ 500 kcal) on “fast” days; 125% on “feast” days vs. CR 75% daily	12 month	Weight -6.8% ADF vs. -6.0% CR; fat mass -5.2 kg both; HDL +6%; LDL -10%; insulin resistance -7%. Adherence lower in ADF (38% dropout).	Confirms ADF ≈ CR for long-term weight loss/metabolic outcomes; adherence is key.
Carter et al., 2016 [162]	63 patients with T2D	2 days/week 70% restriction (~500-600 kcal) vs. daily 25% restriction	12 week	HbA1c -0.8% both groups; FPG -12 mg/dL; no severe hypoglycemia; medication use unchanged.	IF can safely improve glycemia in T2D under supervision.
Harvie et al., 2013 [163]	107 overweight women (BMI ≈ 30)	2 days/week ~500 kcal vs. daily 25% CR	3 months	Weight -5.7% IF vs. -5.0% CR; fat -3.7 kg vs. -3.1 kg; insulin -25%; HOMA-IR -30%.	Demonstrates comparable efficacy with enhanced insulin sensitivity.
Catenacci et al., 2016 [166]	26 adults with obesity (BMI 33 ± 1)	Zero-calorie ADF (36 hour fast/12 hour feed) vs. daily 25% CR	8 week	Body wt -7.1 ± 1.0%; fat -4.2 kg; lean -1.1 kg; fasting insulin -19%; TG -15%.	Demonstrates metabolic safety/feasibility of strict ADF.
Hoddy et al., 2014 [167]	32 obese adults	ADF with feeding early (8 am-2 pm) vs. late (12 pm-8 pm)	8 week	Early-eating group: LDL -10%, HDL +6%, SBP -5 mmHg, weight -3.2 kg (p < 0.05).	Meal timing influences lipid and BP response to ADF.
Solianik et al., 2016 [171]	10 trained lifters	48-hour total fast	2 days	HRV ↓ 15%; reaction time ↑ 10%; mood slightly ↓; glucose stable; ketones ↑ 5×.	Acute 48-hour fasting transiently alters autonomic and neurocognitive function.
Tinsley et al., 2017 [172]	34 trained young men	TRF (8-hour feeding window / 16-hour fast) + RT 3×/week	8 week	Fat mass -1.6 kg; FFM stable; 1RM bench +7%; squat +10%; testosterone -15% (p < 0.05).	TRF preserves muscle/strength; mild endocrine adaptation.

The molecular regulation of muscle health and longevity

Age is a major risk factor for a wide range of pathological conditions. Sarcopenia, the age-related loss of skeletal muscle mass and function, begins in sedentary individuals around age 25. Muscle mass can decline by 10% by age 40 and up to 40% by age 70. Indeed, from age 50 onward, muscle mass declines at a rate of 1-2% per year [173,174]. This loss significantly impairs functional and metabolic performance, maximal strength, and muscle quality [175]. More importantly, the decline in muscle function with age is strongly linked to a lower quality of life, increased frailty, morbidity, and premature mortality [176,177]. Given that sarcopenia affects approximately 40-50% of adults over 80 years of age, it is now recognized as a major geriatric syndrome [178]. Thus, developing and maintaining muscle mass is one of the most vital elements for long-term health and longevity. The balance between anabolic and catabolic processes is the primary regulator of muscle mass, with mTOR being a key driver of anabolic signaling and muscle hypertrophy [49].

Strategies for Muscle Growth and Maintenance

Resistance training is the most effective way to preserve and increase muscle mass and strength. The effectiveness of a training program for maximizing hypertrophy depends on the manipulation of several acute variables [179]. Most programs use dynamic repetitions, including concentric (CON) and eccentric (ECC) actions, with both types of contractions contributing to significant morphological improvements [180,181]. Optimal hypertrophic responses are often achieved with moderate to heavy loads (six to 15 repetition maximum) and moderate volume (three to four sets per exercise) [182,183]. While a 2:1:4 tempo (two seconds CON; one second pause; four seconds ECC) has been proposed to maximize muscle tension [184], shorter rest periods of one to two minutes are generally recommended for hypertrophy-focused programs [185]. Training frequency also plays a key role. Untrained individuals should perform a full-body routine two to three times per week, while advanced lifters may need to train four to six times per week with an upper/lower body or muscle-group split to maximize gains [182].

Dietary Protein and Leucine’s Anabolic Role

To maximize resistance training adaptations in healthy, exercising adults, total daily protein intake should exceed the Recommended Dietary Allowance (RDA) of 0.8 g/kg/day. Meta-analyses and dose-response studies indicate that protein intakes of approximately 1.6 g/kg/day are sufficient to maximize gains in muscle mass and strength, with little additional benefit from higher intakes. Intakes approaching the upper 95% confidence limit (~2.2 g/kg/day) may serve as a practical upper boundary, particularly under conditions of calorie restriction or very high training loads [186].

For adults aged 65 years and older, maintaining adequate protein intake is essential to prevent sarcopenia and counteract anabolic resistance-the age-related reduction in the muscle-building response to dietary protein. Current expert consensus recommends a minimum of 1.0-1.2 g/kg/day of high-quality protein to maintain general health and muscle mass. For older adults who are physically active or engaged in resistance training, a higher intake of 1.2-1.6 g/kg/day is considered optimal for preserving or increasing lean body mass and strength. Evidence does not show additional benefit beyond this range [187,188].

The anabolic quality of a meal is largely dependent on the amino acid leucine. To maximally stimulate MPS, it is necessary to consume enough leucine (approximately 3 g or 0.05 g/kg body weight) to saturate the mTOR signaling pathway [189]. This amount of protein will vary based on the leucine content of the food source; protein-rich sources like dairy, eggs, and meats are generally more effective than leucine-deficient sources like wheat [190]. Whey protein, for example, has been shown to be particularly effective due to its rapid digestion and high leucine content. Consuming whey protein leads to a more significant aminoacidemia than casein or soy protein, with one study showing a 73% higher blood leucine area under the curve (AUC) for whey compared to soy, and 200% higher than casein. This superior amino acid profile from whey protein, especially when combined with resistance exercise, results in a substantially greater protein synthesis response than with casein or soy [191].

Protein Dosing and the Anabolic Response

The anabolic effect of a meal appears to be transient; after a complete meal, both plasma and intramuscular leucine concentrations peak between 45 and 180 minutes, but MPS returns to baseline by 180 minutes. This pattern parallels the phosphorylation of mTOR targets such as 4E-BP1 and S6K, which also peak around three hours and closely track plasma leucine levels [192]. To maximally stimulate MPS after a single resistance exercise session, a dose of approximately 20 g of high-quality protein is generally sufficient in young adults, although studies have shown that 35 g of whey protein can result in greater amino acid uptake and MPS stimulation in older men [193,194]. Consistent with this, evidence indicates that older adults require a higher relative protein intake, around 0.40 g/kg per meal, compared to younger adults (~0.25 g/kg) to achieve maximal stimulation of myofibrillar protein synthesis following resistance exercise (Table 3) [195].

**Table 3:** Principal Human Trials on Protein Distribution/Timing and Acute Anabolic Response Human studies quantifying how dose, type, and timing of protein affect muscle protein synthesis (MPS) and training outcomes. Young adults typically plateau near ~0.25 g/kg (~20-25 g) post-exercise; older adults often require ~0.4 g/kg (≈30-40 g). High-leucine whey outperforms slower/plant proteins acutely; meta-analyses suggest ~1.6 g/kg/day as the practical daily target (with diminishing returns beyond).

Study	Population	Intervention	Duration	Main Findings	Relevance
Morton et al., 2018 [186]	Meta-analysis, 49 RCTs (n = 1,863)	Protein + RT vs. RT	6-52 weeks	Hypertrophy benefit increased with intake up to ~1.6 g/kg/day (upper 95% CI ≈ 2.2); beyond this, diminishing returns.	Practical daily target for trainees.
Nunes et al., 2022 [188]	SR/MA, 105 studies (healthy adults)	Habitual intake strata	Varied	≥1.2 g/kg/day associated with better mass/function vs. <1.0 g/kg/day across ages.	Reinforces >RDA intakes for function.
Paddon-Jones et al., 2004 [189]	6 older women (≈68 years)	15 g EAAs vs. control	3 hours	Net MPS ≈ +120% vs. control despite small dose.	EAAs effective when total volume must be low.
Paddon-Jones et al., 2005 [190]	7 elderly (≈71 years)	15 g whey vs. 15 g EAAs	3 hours	Whey elicited ~50% greater MPS than isocaloric EAAs; faster leucine delivery.	Prioritize leucine-rich whey over isolated EAAs.
Tang et al., 2009 [191]	9 young men (≈20 years)	25 g whey hydrolysate vs. casein vs. soy post-RE	4 hours	Acute MPS: whey +122% > soy +60% > casein +31%; whey = fastest aminoacidemia.	Protein type and digestion rate matter.
Pennings et al., 2012 [193]	48 elderly men (≈73 years)	10, 20, 35 g whey (rest)	6 hours	35 g produced peak leucinemia (≈+250%) and MPS ≈ +75% vs. 10 g; 20 g intermediate.	Supports 30-40 g/meal target in elderly.
Moore et al., 2009 [194]	6 young men (≈24 years)	Whey 0, 5, 10, 20, 40 g post-RE	4 hours	MPS rose dose-dependently and plateaued ~20 g (~0.25 g/kg); 40 g did not add meaningful MPS vs. 20 g.	Defines minimal effective post-exercise dose in young.
Moore et al., 2015 [195]	10 older (≈70 years) vs. 10 younger (≈22 years) men	0.25 vs. 0.50 g/kg post-RE	4 hours	Older needed ~0.40 g/kg to maximize myofibrillar MPS; younger plateaued near 0.25 g/kg.	Quantifies anabolic resistance; higher per-meal dose with aging.
Trommelen et al., 2023 [196]	16 trained men	0, 25, 50, 100 g whey post-RE	12 hours	MPS rose beyond 50 g up to 100 g (area-under-curve), suggesting no strict per-meal “upper limit” within this range.	Cautions against rigid 20-40 g cap; consider context/energy balance.
Witard et al., 2024 [197]	Commentary (on 2023 trial)	Critical appraisal	-	Highlights methodological nuances (e.g., tracer windows, whole-body vs. myofibrillar synthesis) to temper interpretation of “no upper limit.”	Applies guardrails when translating very large boluses.

The 100 g Protein Bolus Study: A Cautious Interpretation

The study by Trommelen et al. in 2023 is noteworthy for demonstrating that ingestion of a large, single bolus of 100 g of protein can produce a prolonged elevation of plasma amino acids and a sustained MPS response lasting over 12 hours [196]. These findings should be interpreted with caution, as the trial involved only young, healthy men, and it remains uncertain whether similar responses would occur in women, older adults, or clinical populations. Importantly, the greater cumulative MPS observed with the 100 g dose was driven by a longer duration of synthesis, not by a higher peak MPS rate, suggesting that large doses extend the anabolic window rather than amplify the maximal anabolic response. A commentary by Oliver C. Witard and Samuel Mettler, along with other expert interpretations, further emphasized that these results should not be taken to imply that there is “no upper limit” to muscle anabolism and that their practical implications have often been overstated or misinterpreted [197].

Practicality and Population-Specific Findings

While the 100g dose did result in a higher cumulative MPS over 12 hours, the initial rate of synthesis was not necessarily higher than with a 25 g dose. The greater overall effect was due to the prolonged duration of elevated MPS, not an increased peak rate. Witard and Mettler suggest that a distributed protein intake (e.g., four 25 g doses) may still be a more effective strategy for maximizing cumulative MPS. Furthermore, a significant portion of the amino acids from the 100 g dose were oxidized and used for energy, rather than being directed solely toward muscle building. A single 100 g protein dose is also not practical for most individuals and can cause gastrointestinal distress. The original study was conducted on recreationally active young men, and the findings may not apply to other populations, such as resistance-trained young women, where other research suggests a more limited anabolic response to large protein doses. Most research still supports the practicality and efficacy of consuming protein in smaller, more frequent doses (20-40 g) throughout the day to maximize long-term muscle growth [196,197].

Intermittent Fasting and Its Catabolic Effects

IF encompasses various eating patterns, including alternate-day fasting, the 5:2 regimen, and time-restricted eating with fasting intervals typically ranging from 12 to 40 hours. Across human trials, IF consistently promotes reductions in body weight and fat mass, which in turn lead to improvements in insulin sensitivity, lipid profiles, and blood pressure [155]. Some studies also suggest weight-independent metabolic benefits, such as enhanced glycemic control and reduced inflammation, although these findings remain inconsistent due to differences in study design and fasting protocols. Mechanistically, fasting induces a metabolic shift that activates AMPK and inhibits mTOR signaling, favoring catabolic energy pathways and enhancing metabolic flexibility [154]. This energetic reprogramming also stimulates ketogenesis and autophagy, processes linked to cellular repair, stress resistance, and longevity. While these mechanisms offer biological plausibility for IF’s potential protective effects against metabolic, neurodegenerative, and malignant diseases, direct evidence of disease-risk reduction in humans remains limited [198].

Limitations

This review is narrative in nature and, therefore, subject to selection bias and variability in the depth of evidence cited. Considerable heterogeneity exists among IF regimens, protein intake studies, and concurrent training protocols, making direct comparisons difficult. Many mechanistic insights derive not only from human research but also from animal and cell-based models, which may not fully capture the complex physiological adaptations occurring in free-living humans. In addition, several studies rely on surrogate molecular endpoints - such as phosphorylation of signaling proteins - rather than long-term functional or clinical outcomes. These limitations warrant cautious interpretation and application of the discussed pathways to exercise and nutrition programming in practice.

## Conclusions

Optimizing muscle growth and long-term metabolic health requires maintaining a dynamic equilibrium between mTOR activation, which stimulates MPS and cellular growth, and AMPK signaling, which supports metabolic efficiency, autophagy, and cellular renewal. Acute activation of mTOR through protein intake and resistance exercise promotes hypertrophy, yet sustained overnutrition and chronic mTOR activity can attenuate AMPK signaling, leading to reduced autophagy, insulin resistance, and impaired cellular maintenance. In contrast, AMPK activation during fasting enhances mitochondrial biogenesis, autophagy, and stress resilience-mechanisms that counter metabolic dysfunction and protect against chronic diseases such as obesity, diabetes, neurodegeneration, and cancer. Across human trials and meta-analyses, total daily protein intakes of approximately 1.6 g/kg/day are sufficient to maximize muscle growth, while up to 2.2 g/kg/day may be advantageous under calorie restriction or heavy training. In older adults, 1.2-1.6 g/kg/day of high-quality protein helps offset anabolic resistance and preserve lean mass. The anabolic potential of a meal is closely tied to its leucine content-about 3 g leucine (≈0.05 g/kg body weight) or 20-40 g of high-quality protein per meal effectively maximizes mTOR activation and MPS.

Building upon the evidence reviewed, the following synthesis represents the author’s conceptual framework for practical application. Sustaining both anabolic and restorative signaling requires a structured rhythm of feeding and fasting. For resistance-trained individuals, 20-40 g of protein every three to four hours effectively stimulates MPS; however, frequent feeding may limit the fasting duration necessary for AMPK activation and autophagy. An 80 kg individual, for instance, would require 128-176 g/day (1.6-2.2 g/kg), achievable with ~43-59 g per meal across three feedings or 32-44 g across four feedings within an eight- to 12-hour eating window, corresponding to 12-16 hours of fasting daily. Strategically integrating higher per-meal protein doses within an eight- to 12-hour eating window (12-16 hours fasting) may optimize both mTOR-driven anabolism and AMPK-mediated cellular renewal. Alternatively, incorporating occasional 24-hour fasts once or twice per week, paired with adequate protein intake on feeding days, could cyclically activate both pathways. While this integrative model remains theoretical, it offers a biologically grounded framework for balancing growth and repair, supporting muscle hypertrophy, metabolic resilience, and healthy longevity, and should be further explored in future human studies.
